# Unveiling global species abundance distributions

**DOI:** 10.1038/s41559-023-02173-y

**Published:** 2023-09-04

**Authors:** Corey T. Callaghan, Luís Borda-de-Água, Roel van Klink, Roberto Rozzi, Henrique M. Pereira

**Affiliations:** 1 German Centre for Integrative Biodiversity Research (iDiv), Leipzig, Germany; 2 Institute of Biology, Martin Luther University Halle-Wittenberg, Halle, Germany; 3 Department of Wildlife Ecology and Conservation, Fort Lauderdale Research and Education Center, University of Florida, Davie, FL USA; 4 CIBIO, Centro de Investigação em Biodiversidade e Recursos Genéticos, InBIO Laboratório Associado, Campus de Vairão, Universidade do Porto, Vairão, Portugal; 5 CIBIO, Centro de Investigação em Biodiversidade e Recursos Genéticos, InBIO Laboratório Associado, Instituto Superior de Agronomia, Universidade de Lisboa, Lisbon, Portugal; 6 BIOPOLIS Program in Genomics, Biodiversity and Land Planning, CIBIO, Campus de Vairão, Vairão, Portugal; 7 Department of Computer Science, Martin Luther University-Halle Wittenberg, Halle, Germany; 8 Zentralmagazin Naturwissenschaftlicher Sammlungen, Martin Luther University, Halle, Germany; 9 Museum für Naturkunde, Leibniz-Institut für Evolutions- und Biodiversitätsforschung, Berlin, Germany

**Keywords:** Ecology, Macroecology

## Abstract

Whether most species are rare or have some intermediate abundance is a long-standing question in ecology. Here, we use more than one billion observations from the Global Biodiversity Information Facility to assess global species abundance distributions (gSADs) of 39 taxonomic classes of eukaryotic organisms from 1900 to 2019. We show that, as sampling effort increases through time, the shape of the gSAD is unveiled; that is, the shape of the sampled gSAD changes, revealing the underlying gSAD. The fraction of species unveiled for each class decreases with the total number of species in that class and increases with the number of individuals sampled, with some groups, such as birds, being fully unveiled. The best statistical fit for almost all classes was the Poisson log-normal distribution. This strong evidence for a universal pattern of gSADs across classes suggests that there may be general ecological or evolutionary mechanisms governing the commonness and rarity of life on Earth.

## Abstract (web summary)

Using occurrence records from the Global Biodiversity Information Facility, the authors show that the global species abundance distribution of 39 taxonomic classes of eukaryotes is best fit by a Poisson log-normal distribution.

## Main

That some species are rare and others are common is one of the oldest observations in ecology. But the exact shape of the distribution of commonness and rarity among species on Earth has remained elusive. Some have argued that nature shows a bias towards rare species^1^, while others have proposed that most species have intermediate abundances^2^. Accordingly, different statistical distributions have been proposed as a model of the distribution of species abundances, including the log-series distribution^1^ (corresponding to a monotonic decrease of the number of species with increasing species abundance) and the log-normal distribution^3^ (corresponding to a unimodal distribution of the number of species along the abundance axis in log-scale). In addition, Preston proposed that, at low sampling efforts, the log-normal distribution seems like a monotonically decreasing function because of the presence of a ‘veil line’^3^, since most species will occur at densities below the detection threshold. The existence of such a veil line, or its generality, has been questioned^4,5^, while others have suggested it does exist^6,7^. Regardless, these different models, and their corresponding conclusions, have important consequences for biodiversity research and conservation^8^ as well as for estimating the number of species on the planet^9^. Understanding if a universal shape of species abundance distributions (SADs) exists may help illuminate how life on Earth is maintained.

Who can explain why one species ranges widely and is very numerous and why another allied species has a narrow range and is rare? — Darwin, *On the Origin of Species* p. 21 (1859)^10^

Both the log-series and the log-normal models were mostly phenomenological or, at best, tried to capture a statistical sampling process. More recently, ecological and evolutionary mechanisms (such as species’ interactions, migration and speciation) that may drive SADs have been examined using theory^11,12^. For instance, it has been shown that a simple birth–death process results in a negative-binomial distribution that approaches the log-series distribution under certain conditions and under other conditions it approaches the unimodal shape of the log-normal distribution^9^. Other mechanisms that have been suggested to lead to a log-normal SAD include random multiplicative interactions between species^13^ and niche partitioning models^14^.

**Fig. 1:**
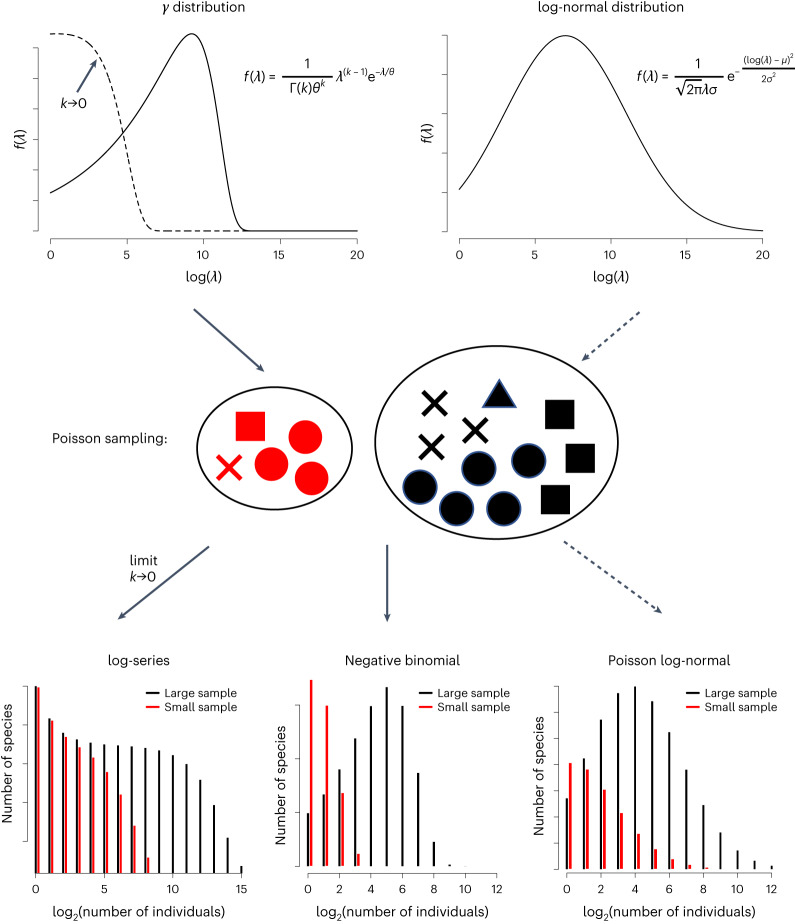
Conceptual scheme illustrating the Poisson sampling of a community with species abundances described by a gamma or a log-normal distribution. Two types of gSAD—gamma (left) and log-normal distribution (right) are shown at the top. Each distribution represents the probability *f* of a species having a given abundance *λ*, with the gamma distribution having parameters *k* (shape) and *θ* (scale) and the log-normal distribution having parameters *μ* (mean) and *σ* (standard deviation), and Γ() representing the gamma function. In the middle, sampling of the gSAD with the probability of each species having a given number of individuals sampled described by a Poisson distribution is illustrated. The mean abundance of each species sampled is randomly taken from the SAD. We exemplify two samples of different sizes, where different symbols denote individuals of different species. The bottom graphs show that: if the global abundances have a log-normal distribution, the mixture distribution of abundances in the sample is a Poisson log-normal; if the global abundances follow a gamma distribution the resulting mixture distribution is a negative binomial but in the limit *k*→0, we obtain the Fisher log-series.

Species in a biological sample (compared with those from fully quantified communities) are the result of statistical sampling of an underlying SAD and this sampling process can be used to unify three proposed statistical distributions. The negative-binomial distribution corresponds to Poisson sampling of an underlying gamma distribution^15^, with the log-series corresponding to a particular case of the gamma distribution where the shape parameter tends to zero. A Poisson log-normal distribution results from sampling an underlying log-normal distribution^14^. For smaller samples, a phenomenon similar to a veil line occurs, whether the underlying SAD follows a log-normal or a negative-binomial distribution (Fig. 1). The log-normal and gamma distributions are two of the top candidates to understand the SAD as there is empirical and theoretical support for both distributions^9,14,15^ and the gamma distribution is particularly flexible, encompassing both unimodal distributions with varying skewness and monotonically decreasing distributions. Importantly, the sampled SAD may qualitatively differ from the underlying SAD.

Despite decades of research and dozens of proposed statistical fits to describe SADs^16^, there remains little conclusive evidence for the shape of SADs (compare refs. ^6,17–19^). The debate surrounding the shape of SADs may be partly driven by the fact that the empirical data on which these distributions are fitted has historically been focused on local-scale biodiversity samples^20^. Local communities are often investigated as natural assemblages but are subject to many idiosyncrasies, such as species that are common in some parts of their range but rare in other parts of their range^21^, species that move in and out of a location throughout the year (for example, migratory birds^22^) or species detected that are vagrant individuals from adjacent ecosystems. Such idiosyncrasies can influence the shape of a SAD^22^. This problem may be overcome by using synthesis approaches, looking at many different datasets at once (for example, refs. ^16,23^) or by using data at the global scale^24,25^, since in such a ‘closed’ system, local-scale immigration and emigration effects can be excluded. Hence, at a global scale, the SAD may not represent assemblages of ecologically co-occurring species but may be able to reveal evolutionary processes such as the dynamics of speciation. Nevertheless, there remain many challenges with using global-scale data to quantify a SAD, as fully sampling the global flora or fauna is a massive undertaking^24^. Quantifying a global species abundance distribution (gSAD) could advance the understanding of rarity but at the global scale, minimizing potential problems of measuring rarity at local scales. Further, assessing SADs can potentially (1) advance the testing of ideas about the processes underlying the generation of rare species, (2) assess universality in mechanisms of speciation across different taxonomic groups (for example, classes) and (3) provide insights to better understand how anthropogenic changes (for example, climate change), which often occur at large scales, can influence rarity.

Here, we leverage the largest biodiversity aggregator of global biodiversity records—the Global Biodiversity Information Facility (GBIF)—to assess the shape of the gSAD and how it varies among taxonomic groups. GBIF has aggregated data at a vastly broader geographic, taxonomic and temporal scale than previously available and has done so at an accelerating rate. We downloaded data from GBIF from the period 1900 to 2019, representing a total of ~1.38 billion occurrences of species across 39 taxonomic classes (Supplementary Fig. 1), to quantify the shape of the gSAD. For each taxonomic class, we calculated a gSAD using a 20-year rolling window for each year from 1900 to 2000 (Methods), by aggregating the number of occurrences in GBIF for each species belonging to that taxonomic class. This approach assumes that the number of observations in GBIF is a proxy for the relative abundance of a species in the world (sensu ref. ^24^; Methods), which we have verified to be a good approximation at least for birds (Supplementary Figs. 2 and 3). In our work, rarity is presumed as a function of the number of occurrences available in GBIF. On a linear scale, most species are still rare as they are represented by only few occurrences.

## Results and discussion

**Fig. 2:**
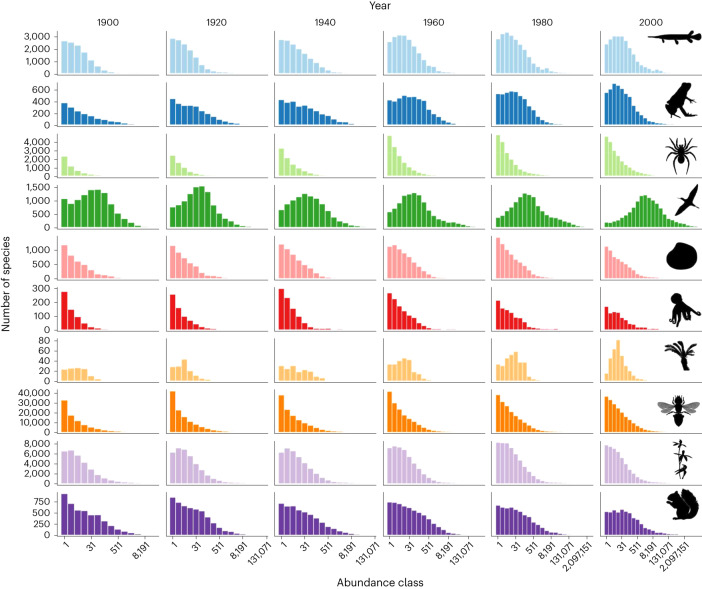
The temporal evolution of the gSAD. From top to bottom: Actinopterygii, Amphibia, Arachnida, Aves, Bivalvia, Cephalopoda, Cycadopsida, Insecta, Liliopsida and Mammalia. For some classes, the apparent unveiling is evident, such as for Aves. Each year represents a rolling 20-year window in which GBIF observations were aggregated.

Our analysis shows that as global biodiversity sampling increases through time (Supplementary Fig. 4) the shape of the gSAD is unveiled, that is, the qualitative shape of the sampled gSAD changes revealing the underlying gSAD. This is most evident for some well-sampled taxa such as birds (Fig. 2), where by about the year 2000 the entire distribution is uncovered showing a unimodal distribution of abundances with log-left skew^25^. For other classes, the entire distribution is not yet uncovered but similar patterns of ‘unveiling’ can be seen for Amphibia, Cycadopsida and Mammalia. In contrast, for some classes (for example, Insecta) we see that the veil is not uncovered and the qualitative shape of the gSAD remains monotonically decreasing (Supplementary Videos for all 39 classes). Even when sampling is not aggregated across multiple years and each year is treated independently, the veil is lifted for birds (Supplementary Fig. 5), indicating that in each individual year, the complete gSAD for birds is currently being sampled. In other words, nearly all species of birds are being sampled annually.

**Fig. 3:**
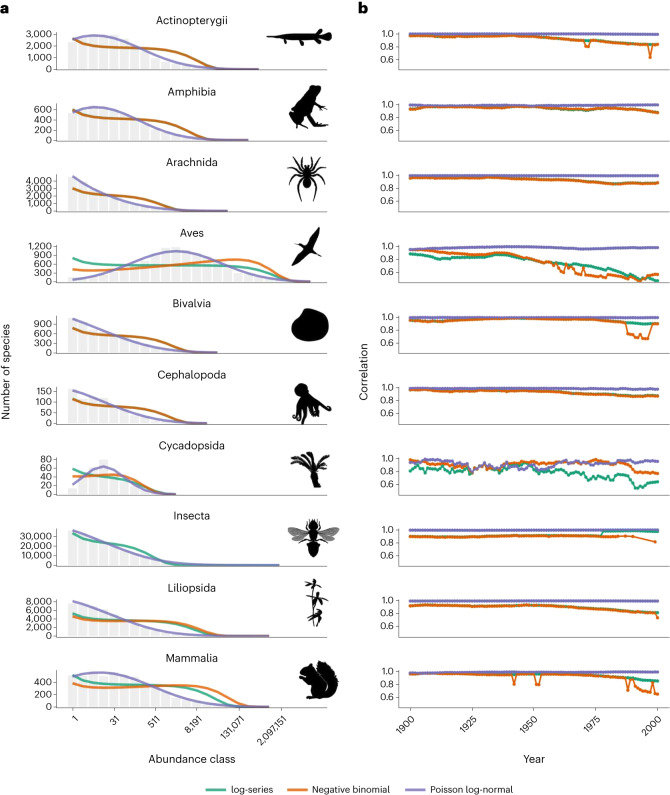
The temporal change in our statistical understanding of gSADs. **a**, The final 20-year rolling window gSAD for each of ten example classes with the best fit overlaid for the log-series, negative binomial and Poisson log-normal distributions. **b**, Yearly goodness of fit (correlation) of each distribution for each 20-year rolling window gSAD. Example classes from top to bottom: Actinopterygii, Amphibia, Arachnida, Aves, Bivalvia, Cephalopoda, Cycadopsida, Insecta, Liliopsida and Mammalia.

A more important biological question is: what is the underlying shape of a gSAD? To answer this question, maximum likelihood estimation can be used to tease apart the difference between the observed shape of a gSAD and the underlying distribution from which that gSAD was sampled^14,26^. By assessing the statistical distribution of the underlying gSADs we can draw inferences about if, and to what extent, taxonomic classes have similar ecological and evolutionary processes that underlie the pattern of SADs. We tested the statistical fit of the empirical distributions (Fig. 2; Methods) and found that, for 38 out of 39 classes, the statistically best fit of the three distributions was the Poisson log-normal (Supplementary Fig. 6). This suggests that there may be universality in the shape of a gSAD across taxonomic groups. Importantly, the evidence base shifts temporally, where early in the time series there is more uncertainty as to which distribution provides the best fit, but it is clear that Poisson log-normal provides the best fit by the end of the time series for nearly all classes analysed (Fig. 3 and Supplementary Fig. 6). The evidence for better fit of the Poisson log-normal was greater in better sampled groups where the mode of the distribution had been unveiled. But even for groups where we are far from unveiling the mode, such as insects, the Poisson log-normal still fits the data best using maximum likelihood estimation. In addition, when one examines within Insecta, the two best-sampled and relatively well-known groups—dragonflies and butterflies—the gSAD shape qualitatively appears more log-normal-like than for Insecta as a whole (Supplementary Figs. 7 and 8). Additionally, some of the most speciose insect orders (Diptera and Coleoptera) showed strong statistical support for a Poisson log-normal distribution, despite presumed differences in speciation rates (Supplementary Figs. 9 and 10).

**Fig. 4:**
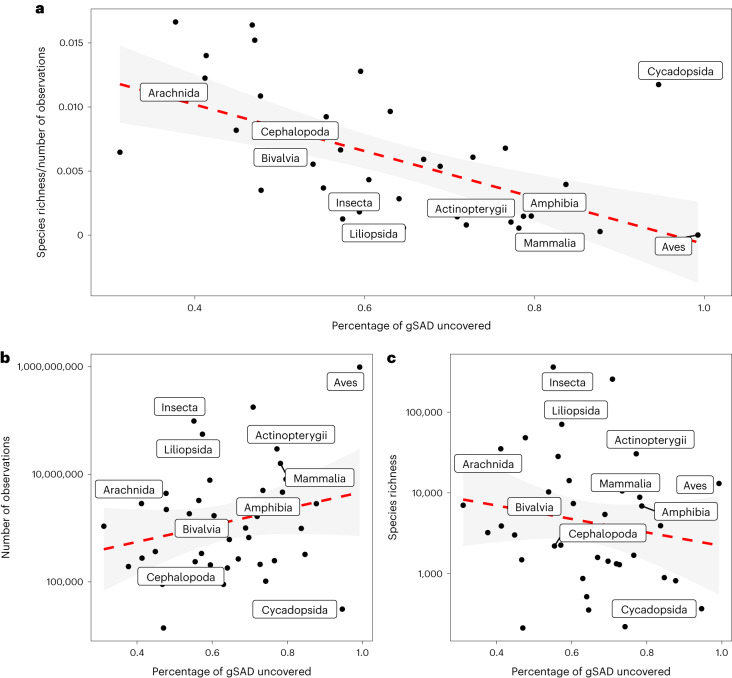
How the relative position of the veil corresponds to species richness and the number of individuals in a class. **a**–**c**, The proportion of the gSAD uncovered, assuming a Poisson log-normal distribution, and its relationship to observed species richness/number of observations (**a**), number of observations (**b**) and species richness (**c**). To aid in visualizing the patterns, the red dashed line represents a fit from geom_smooth() and the shaded grey area represents the 95% confidence interval around that fit.

The relative position of the veil provides an assessment of how well the species richness of that group has been described. The fraction of species unveiled can be expected to depend on the total number of species in the group and the number of individuals sampled^19^. As we do not know the true number of species in most groups, we used the observed species richness to examine its influence on the position of the veil. We found that the percentage of the gSAD that is unveiled is strongly dependent on observed species richness, where more speciose classes are less well-sampled (parameter estimate = −0.04, 95% highest-density interval (HDI) = −0.11, 0.03; Fig. 4 and Supplementary Fig. 11), as well as sampling effort, where an increased number of occurrences allows for a higher likelihood of having the class fully sampled (parameter estimate = 0.04, 95% HDI = −0.01, 0.09; Fig. 4 and Supplementary Fig. 12). The position of the veil was also strongly negatively related with the proportional species sampling, obtained by dividing the observed number of species by the number of occurrence records (parameter estimate = −0.17, 95% HDI = −0.24, −0.10; Fig. 4 and Supplementary Fig. 12). This analysis also suggests that, while most species of groups such as birds and cycads have been described and mobilized to GBIF, at least half of the species of other groups such as arachnids and insects remain to be discovered and/or mobilized to GBIF. It is important to highlight that this is probably an underestimate of how many species remain to be discovered and mobilized, as the species richness estimates based on the veil of the log-normal distribution can underestimate the real number of species^15^. Future work should look to further refine methods to estimate species richness on the basis of the position of the veil of the log-normal distribution. As some of the taxonomic groups with the least unveiling of their gSAD are also the most speciose taxa, it seems that to take stock of the total diversity of species on the planet, we need to increase both the rate of species description and the mobilization of data.

Our results illustrate two key points about our empirical understanding of SADs. First, we show that there is indeed a veil line in SADs (compare ref. ^17^), in agreement with previous theoretical results^6^. Second, our ability to see this veil is dependent on sampling effort^7^ and, using time as a proxy for sampling effort, we show that the veil line is ‘lifted’ as we continuously increase our knowledge of global biodiversity through time. This suggests that care must be taken in extrapolating the overall shape of a SAD just from a sample of individuals. There are now many approaches for upscaling SADs from small samples to the full community of interest^5,9,27–29^. However, they usually require knowledge about the shape of the SAD of the full community, which is not always known and often needs to be inferred from the sampled SAD. This may lead to erroneous conclusions when the sample is small, as the power to discriminate the fit of the observed distribution to different probability distributions increases with sample size (Fig. 3b).

We provide strong evidence that the shape of the gSAD seems to be well approximated by a Poisson log-normal distribution across many taxa. Our results are consistent with recent findings at the global scale for land plants^24^ and birds^25^. This contrasts with a recent review at a non-global scale that has found that the log-series was the best fit across many different SAD datasets, albeit support for Poisson log-normal and negative binomial was also high^16^. Other studies find that support for log-normal may increase with spatial scale and that log-series only fits observed SADs at the local scale^20,30^. One taxonomic group where log-series^31^ or negative binomial^15^ were thought to be the best fitting distribution, at least at the regional scale of the Amazon, is trees^31^. However, a specific test for this group (Methods) showed strong support for a Poisson log-normal at the global scale (Supplementary Fig. 14). Therefore, despite interest on invariance of SADs across spatial scales^9,28^, it may well be that subglobal SADs differ from global SADs beyond the sampling mechanism modelled by Poisson sampling. The dominant ecological processes operating at different spatial scales are distinct^27,30^ and modelling the spatial scaling of the SAD may require the understanding of the ecological processes that determine the spatial aggregation of species and their interactions^5^.

Our results are largely descriptive and empirically focused and our study was not designed to disentangle the mechanistic and stochastic processes that can lead to a SAD. But nevertheless, our finding of the ubiquitous Poisson log-normal SAD shape across taxonomic classes invites some speculation. According to neutral theory, point mutation leads to a log-series SAD while random fission leads to a unimodal SAD^12^, while a log-normal SAD is recovered under a ‘broken stick model’ where a part of a ‘stick’ is broken independently of its size^14,32^. The random fission model is often associated with allopatric speciation. In addition, it has been shown that even point mutation can lead to unimodal SADs when new species are not recognized for some generations, that is, protracted speciation^33^. Therefore, we speculate that allopatric speciation and/or protracted speciation could be a dominant mode of speciation at the global scale and across taxonomic groups. However, a log-normal gSAD can result from many different mechanistic or stochastic processes. For instance, it has been argued that the log-normal distribution at large spatial scales may result from splicing SADs from contiguous plots in a statistical convergent process analogous to the central limit theorem^29^. Further testing would be needed to uncover if, and to what extent, our results indicate that there is a dominant mode of speciation at the global scale.

## Future avenues of exploration

The results presented here illustrate the importance of considering SADs in a global context but also highlight the importance of future work to better understand the shape of gSADs. In this analysis, we contrasted taxonomic classes but we recognize that these are somewhat arbitrary units and that there is a substantial amount of ecological and evolutionary variation within and between classes. Species could be contrasted in different ways (see refs. ^23,34,35^), for example, by speciation rate, body size, feeding types or at lower phylogenetic branching levels than class—all of which could form future work when data from GBIF are integrated with external datasets. Also, the species concept on which our analysis, and indeed all of GBIF, is based could be debated and has probably changed over time as our technological and empirical capacity to separate species has grown^36,37^. Our analysis assumed a Poisson sampling process with uniform spatial sampling of random (non-aggregated) species distributions^5^. We know that both assumptions are not necessarily upheld in our data, as species are aggregated and GBIF data are not globally uniform (for example, there are biases towards temperate and built-up regions of the world^38^) but we believe our work offers a starting point to investigate how potential aggregations of species distribution can influence the shape of a gSAD. We also focus on a ‘log-normal-like’ shape of a distribution, not necessarily investigating the intricacies of the shape of the gSAD such as log-left skew^18,25^. Our assumption of proportionality in the number of observations in GBIF in relation to the species global abundance is not sufficiently exact to assess this level of detail in the shape of the gSAD and this is an issue that certainly merits more research.

GBIF is increasingly aggregating larger amounts of data, driven in part by contributing citizen science participants. Nevertheless, there exist biases in data representation of GBIF^38^. Such biases probably have the potential to influence the relative position of the veil, for example by leaving insects further away from being unveiled given that insects are most diverse in the tropics where they are unlikely to be fully sampled. However, we believe GBIF offers a rich source of future analyses investigating SADs and/or gSADs. For example, future analyses could look at different regional samples, where other ecological (for example, dispersal) and evolutionary processes (for example, speciation rates) are potentially driving patterns in the SAD. Yet, one limitation, at present, is that GBIF is dominated by presence-only data, which is why we used the number of occurrences as a proxy for abundance (sensu ref. ^24^). How, and the extent to which, the number of occurrences in GBIF correlates with true abundance is an important field of study in the future as GBIF data are increasingly used to answer ecological questions (Methods).

## Conclusions

Our work used global biodiversity data from GBIF to empirically and statistically illustrate the gSAD. We show that there is undoubtedly a veil that is lifted when sampling effort increases. Importantly, with sufficient sampling, as is the case for birds, there may be the possibility to use large-scale datasets such as GBIF to track global biodiversity change through time. Our results also suggest that there may be some universality in the shape of the gSAD. Worryingly, the way that humans are changing the abundance of different species, for instance by making common species less common and homogenizing species across the planet, may have implications for gSADs in the future. It is our hope that as the global community continues to increase our knowledge of biodiversity (for example, through the mobilization of data), so too will we continue to unveil the diversity of our planet and understand how anthropogenic changes are altering those patterns.

## Methods

### Overview

We used data from the GBIF to calculate gSADs for each class of eukaryotic organisms (*n* = 39) included in the analysis. First, we downloaded all data on GBIF and aggregated the total number of observations (occurrence records) for each species included in GBIF. We then empirically summarized these aggregations and how these changed through time, providing a time series of the gSAD. We used a temporal component in our analysis as it represents the evolution of the global understanding of biodiversity, using the best-available dataset to do so—GBIF. As the number of records increases with time, this allowed us to approximate the potential knowledge about the shape of the gSAD. We acknowledge that time is only one way to investigate the evolution of a gSAD but time was chosen in part to make the analysis computationally tractable, avoiding pure resampling analyses that could quickly become computationally expensive. We feel it is useful in this case to advance understanding of the shape of the gSAD. We used SAD sampling theory to assess whether the statistical shape of the observed gSAD corresponded to one of three potential distributions: log-series, negative binomial or Poisson log-normal. We then used models to quantify the influence of species richness and the number of individuals on the relative position of the sampling veil (sensu ref. ^3^), that is, the percentage of species in the gSAD that have been sampled.

### GBIF data

GBIF is the world’s largest biodiversity aggregator, housing >2 billion biodiversity records with a 12-fold increase in available data since 2007^39^ (Supplementary Fig. 4). We downloaded GBIF data on 4 February 2021^40^. Data were downloaded as a .avro file and processed using SQL in Google BigQuery. All GBIF records were aggregated by year and by species, providing a list of ‘abundance’ for each species in each year. This approach assumes that the total number of observations (occurrences) in GBIF is a proxy for the actual abundance of a species in the world^24^. To test this assumption we used birds, the only taxonomic group for which for the most species abundance estimates exist and correlated the number of GBIF records for each species with the estimated global abundance of birds from ref. ^25^ (*n* = 9,047 species) and from BirdLife International (*n* = 3,216 species). In both instances, correlation was relatively strong (Supplementary Figs. 2 and 3) suggesting that, indeed, the number of occurrences in a global database may serve as a proxy for the actual abundance in the world. See section on ‘Assessing the sensitivity of using the number of occurrences as a proxy of abundance’ for more.

Our analysis was performed at the class level and after downloading GBIF we used some minimum criteria to select those classes for potential inclusion in our analyses. To be included, a class needed to have at least 10 observations per year, at least 200 total species and, on average, 50 observations per species. We analysed a total of 39 classes for which SADs were assessed (Supplementary Fig. 1).

### Visualization of gSADs

We empirically summarized the observed gSAD for each year for each taxonomic class and visualized these as animated gifs to understand how the qualitative shape of the gSAD changes through time. We used histograms with logarithmic classes of base 2 (octaves), delimited as follows: one individual, two to three individuals, four to seven individuals and so on. There are other ways of delimiting the octaves (for example, ref. ^41^) but the one we adopted here has several advantages. For instance, the boundaries in a logarithmic scale ([log_2_(1) = 0, log_2_(2) = 1[, [log_2_(2) = 1, log_2_(4) = 2[, [log_2_(4) = 2, log_2_(8) = 3[et seq.) are equally spaced and, importantly, it guarantees that the log-series distribution is always a monotonically decreasing curve.

For each class we aggregated our abundance data to create a gSAD in four different methods: (1) aggregation of individual years, where each year is treated as an independent sample; (2) cumulative aggregation across years, where observations are aggregated across years cumulatively starting with year 1900; (3) a 10-year rolling window, where observations are aggregated in 10-year periods using a rolling window; and (4) a 20-year rolling window where observations are aggregated in 20-year periods using a rolling window. The evidence for Poisson log-normal was robust across the different aggregations. We used these different aggregations to examine the advantages of increasing the number of observations in each time window versus the problems that arise when combining data collected many years apart with potentially different sampling and classification methods. Exploratory analyses showed that the number of singletons was artificially high in the early periods and while some of these may be due to real biology (that is, rare species), in general these may also be due to ‘mistakes’ such as misspellings, species names that do not match, changing taxonomy or similar errors that GBIF data is prone to^42^. When using method 2 above, which maximizes the number of observations used in any year by cumulatively including all previous years, this singleton problem is exacerbated. Method 1 is less vulnerable to this problem but uses a limited set of data for each year (only the observations of that year), while methods 3 and 4 combine data from several years (10 and 20 years, respectively) but avoid combining data from years that are too far apart.

Exploratory analysis also showed that when minimizing the number of species to those that taxonomically match accepted taxonomic status for birds (Supplementary Fig. 15) and mammals (Supplementary Fig. 16) similar qualitative and quantitative results are found, with the Poisson log-normal remaining the best statistical fit. In fact, when trimming the species in GBIF to only those that match with an approved taxonomy, the Mammalia distribution appears qualitatively even more log-normal-like. To perform these approved taxonomy-based analyses, we used the Clements taxonomy (https://www.birds.cornell.edu/clementschecklist/download/) and the American Society of Mammalogists Database (https://www.mammaldiversity.org/). This trims the number of species to a lesser number GBIF. We performed this analysis for the last 20-year rolling window only. And we chose Aves and Mammalia as they have two of the most comprehensive and accessible up-to-date taxonomies. To illustrate the potential hazards of GBIF name changes, in the GBIF data we downloaded, in an exploratory analysis of all entities labelled as ‘species’ (~1.5 million), about 1,614 had more than three words (that is, more than simply genus and species), which could include known hybrids and other varieties.

### gSAD distribution fitting

For each observed gSAD (year × class combination) we fit probability distributions for abundances of species in the assemblage using maximum likelihood estimation. Models were fit using the sads package in R^43^. Models were fit on the raw data (that is, the vector of ‘abundances’ where abundances were the number of occurrences in GBIF) and binning was only done for visualization purposes (see earlier for details on visualizations). For each observed gSAD and aggregation as described above we fit three probability distributions: (1) log-series; (2) negative binomial; and (3) Poisson log-normal (Fig. 1). For details and procedures of the statistical fits, see the reference material located in ref. ^37^, available at https://cran.r-project.org/web/packages/sads/sads.pdf. The negative-binomial distribution was fit using a truncation at zero. Starting values were necessary for the maximum likelihood estimation of the negative-binomial distribution. For this, we used the mean number of observations across species and an estimate of the shape parameter of the corresponding gamma distribution based on the mean and variance of observations across species (*k* = mean^2^/(variance − mean)). We tested the sensitivity of the starting parameters of the negative-binomial distribution by using many starting parameters, creating a vector of 100 values from ±20% for the mean and ±20% for *k*. For illustrative purposes we did this for both the individual years (method 1 above) and cumulative (method 2 above) aggregation types for the years 1925, 1950, 1975, 2000 and 2018 for Aves, Amphibia, Arachnida and Mammalia. Visual inspection shows that the predicted fits were similar regardless of the starting parameter values (Supplementary Figs. 17–20).

### Goodness of fit

To quantify the statistical likelihood of a given distribution (log-series, negative binomial or Poisson log-normal) representing the observed gSAD we used Pearson correlation^23^. We used the observed values for each abundance class and compared these with the predicted values for each abundance class, where the predicted values were derived from the statistical fitting of the gSAD, described above. In the main text, we report the Pearson correlation value as a measure of goodness of fit (Fig. 3). However, we also show that other measures of goodness of fit (*χ*^2^ value, Kolmogorov–Smirnov *D* statistic and Kolmogorov–Smirnov *P* value) are strongly correlated and provide qualitatively similar results to those presented in our main text, using four illustrative classes (Supplementary Figs. 21–24). Although not a direct measure of goodness of fit, we also used Akaike information criteria to compare the three model fits for the 20-year rolling window for the last year of the time series (2000). This found support that, for nearly all classes, Poisson log-normal was the best fitting model (Supplementary Table 1).

### Testing of trees

We used the above methods and applied them to tree species in the GBIF dataset. To subset all of GBIF data to just trees, we used the list of tree species downloaded from the Botanic Gardens Conservation International global tree list^44^. They use the IUCN Global Tree Specialist Group definition of a tree, defined as ‘a woody plant with usually a single stem growing to a height of at least two meters, or if multi-stemmed, then at least one vertical stem five centimeters in diameter at breast height’. We only included species names that taxonomically matched the global tree list (*n* = 39,065 species). For presentation purposes, we only presented the final year (2000) 20-year rolling window and the trend in correlation of observed and predicted values for all rolling window years from 1900 to 2000.

### Testing of finer taxonomic groups within Insecta

Similar to above, with trees, we performed exploratory analyses by repeating the main analyses but at a finer taxonomic level (dragonflies, butterflies, Diptera and Coleoptera) within Insecta. We chose the first two groups as they are well known and popular taxonomic groups to test if Insecta could show a qualitatively more log-normal-like shape. For dragonflies, we filtered GBIF records to order Odonata whereas for butterflies we filtered GBIF records to the families Papilionidae, Pieridae, Lycaenidae, Riodinidae, Nymphalidae and Hesperiidae. We chose Coleoptera and Diptera because they have a different number of species but are less well known than dragonflies and butterflies, as well as with presumably different speciation rates. For presentation purposes, we only presented the final year (2000) 20-year rolling window and the trend in correlation of observed and predicted values for all rolling window years from 1900 to 2000.

### Modelling the percentage of the gSAD uncovered

For each taxonomic class we estimated the position of the veil, or the proportion of the gSAD uncovered, by 1 − *P*(*X* = 0 | *X* ∼Poisson log-normal(*μ*,*σ*)) where *μ* and *σ* are the fitted parameters of the Poisson log-normal *X*. We used the Poisson log-normal distribution fit for this as this was the superior fit as evidenced above. This value, the promotion of the gSAD uncovered, could theoretically range from 0 to 1 where values close to 1 would indicate that the veil was nearly uncovered and values close to 0 would indicate that the veil was far from being uncovered.

We then used the observed species richness and the total number of observations, across the entire time period (1900–2019) for each taxonomic class as predictors of the percentage of the gSAD uncovered. We also used a proportional value where the observed species richness was divided by the total number of observations to represent a standardized species per effort in a taxonomic class. To quantify the relationship between these three values we fit Bayesian linear regression models where the response variable was the percentage of the gSAD uncovered and the predictor variables were log_10_-transformed. We used brms^45^ for model fitting and tidybayes^46^ for visualization of the posterior distribution. Models were fit with a Gaussian error distribution, default priors, 4 chains, 4,000 iterations and a warmup of 1,000.

### Assessing the sensitivity of using the number of occurrences as a proxy of abundance

We used the number of occurrences in GBIF as a proxy for abundance, where the number of occurrences is treated as a relative measure of global abundance. A similar approach has been used before by ref. ^24^ and allows for different types of data to be aggregated (for example, abundance and presence-only estimates or citizen science and museum-based collections). This results in a relative abundance estimate that can be less biased than local plot-scale abundance data that does not sample a large portion of the world’s surface area^24^. We found strong correlation (*r* ranges from 0.69 to 0.76) between published estimates of absolute abundance for birds and the number of GBIF occurrences for birds (Supplementary Figs. 2 and 3), the only taxonomic class for which such estimates of global abundance per species have been attempted. Yet, we acknowledge that our findings about the universality of the Poisson log-normal distribution fit rely on the number of occurrences being a good proxy for the relative abundance of organisms on the planet and could potentially be affected by biases in the GBIF occurrence data. Since 2010, at least 80% of the data in GBIF have been contributed by some form of citizen science participation^47^. This could lead to a bias towards rare species given a preference for rarity and for citizen science participants to seek out rarity^48^, for example birdwatchers preferentially seeking out rare individuals that could be ‘counted’ multiple times in GBIF. Interestingly, historical museum collections could also exhibit such biases due to a focus of many museum trips in documenting unique or new species^49^. Alternatively, there could be a bias towards common species as these are easiest to observe and document by the public. Although empirical evidence suggests that most citizen science participants report all species they see with no preference for common or rare species^50^, there remains a detectability issue as species that are harder to identify may be under-reported^51^.

We examined the sensitivity of our analysis to such biases using simulations. We assume that the relationship between observed and real abundances can be described by a power law, *λ*_s_ = *p* × *λ*^*q*^, where *λ*_s_ is the observed abundance, *λ* is the global abundance and *p* and *q* are parameters (Supplementary Fig. 25). Biases towards rare species correspond to having *q* < 1, while biases towards common species correspond to *q* > 1. A perfect linear response is represented by *q* = 1. The number of occurrences in GBIF is then assumed to follow a Poisson sampling with mean *λ*_s_. We tested a range of *q* values from 0.1 to 2. The parameter *p* was chosen to ensure that both the biased and non-biased samples had approximately the same number of individuals. We assumed that real species abundances follow a gamma distribution and test whether Poisson sampling would result in the correct SAD being fit (a Poisson sampling of a gamma distribution should result in a negative binomial or log-series). We used the gamma distribution because it captures both the log-series (in the limit of *k*→0) and the negative-binomial SADs. For each value of *q*, we sampled 100 communities with 10,000 species each from a gamma distribution of abundances. We then fit both the log-normal and the negative-binomial distributions to the observed SAD of each community and compared the Akaike information criterion score of both models. Our simulations showed that the Poisson log-normal is the best fit when the number of occurrences is biased towards common species (Supplementary Fig. 26) and that the negative binomial should be the best fit when there are strong biases towards rare species.

We found no evidence to suggest that there was a bias towards common species in GBIF but instead that there might be a bias towards rare species in GBIF, as a log–log regression of the occurrences against estimated abundances exhibits a *q* of around 0.5 (Supplementary Fig. 27) and, as discussed above, this will favour the negative binomial and not a Poisson log-normal fit. So, we conclude that it is unlikely that biases on occurrence data are driving our results of better fits of the Poisson log-normal. We acknowledge that we only tested this with birds as this is the only taxonomic group for which there exists the potential to test this. Quantifying the biases in GBIF for other taxonomic groups remains an important future analysis step as GBIF data are increasingly used in ecological and biodiversity research.

### Data analysis

We used the R statistical software^52^ to carry out our analyses while also relying heavily on the Tidyverse^53^.

### Reporting summary

Further information on research design is available in the Nature Portfolio Reporting Summary linked to this article.

### Supplementary information

Supplementary Information Supplementary Figs. 1–27, Table 1 and captions for Videos 1–39.

Reporting Summary

Supplementary Videos Supplementary Videos 1–39. Animation of the evolution of the gSAD, using 20-year rolling windows, for each of the 39 taxonomic classes included in the analysis.

## Data Availability

All data used for our analyses are freely available from the GBIF (www.gbif.org). The doi representing our download is: 10.15468/dl.4dcbgt. All other auxiliary datasets used (list of tree species, bird abundance estimates and mammal and bird taxonomy lists) are described in the Methods. The animated gifs of gSADs through time for all 39 taxonomic classes can be found in the Supplementary Videos.

## Code availability

Code (and some processed data) to support our analyses are available here: 10.5281/zenodo.8043678.
