# Supplementary material for: Unveiling global species abundance distributions
Source: Nat Ecol Evol. 2023 Sep 4;7(10):1600–9. doi: 10.1038/s41559-023-02173-y (PMC10555817; doi:10.1038/s41559-023-02173-y)
Supplement: Supplementary file 1 — Supplementary Figs. 1–27, Table 1 and captions for Videos 1–39. [file 41559_2023_2173_MOESM1_ESM.pdf]

nature ecology & evolution

Article

https://doi.org/10.1038/s41559-023-02173-y

Unveiling global species abundance distributions

In the format provided by the authors and unedited

Supplementary Materials for

Unveiling global species abundance distributions

Corey T. Callaghan[1,2,3*], Luís Borda-de-Água[4,5,6], Roel van Klink[1,7], Roberto Rozzi[1,8,9], Henrique M. Pereira[1,2,4]

Correspondence to: callaghan.corey.t@gmail.com

This PDF file includes:

Figs. S1 to S27
Table S1
Captions for Movies S1 to S39

Other Supplementary Materials for this manuscript include the following:

Movies S1 to S39

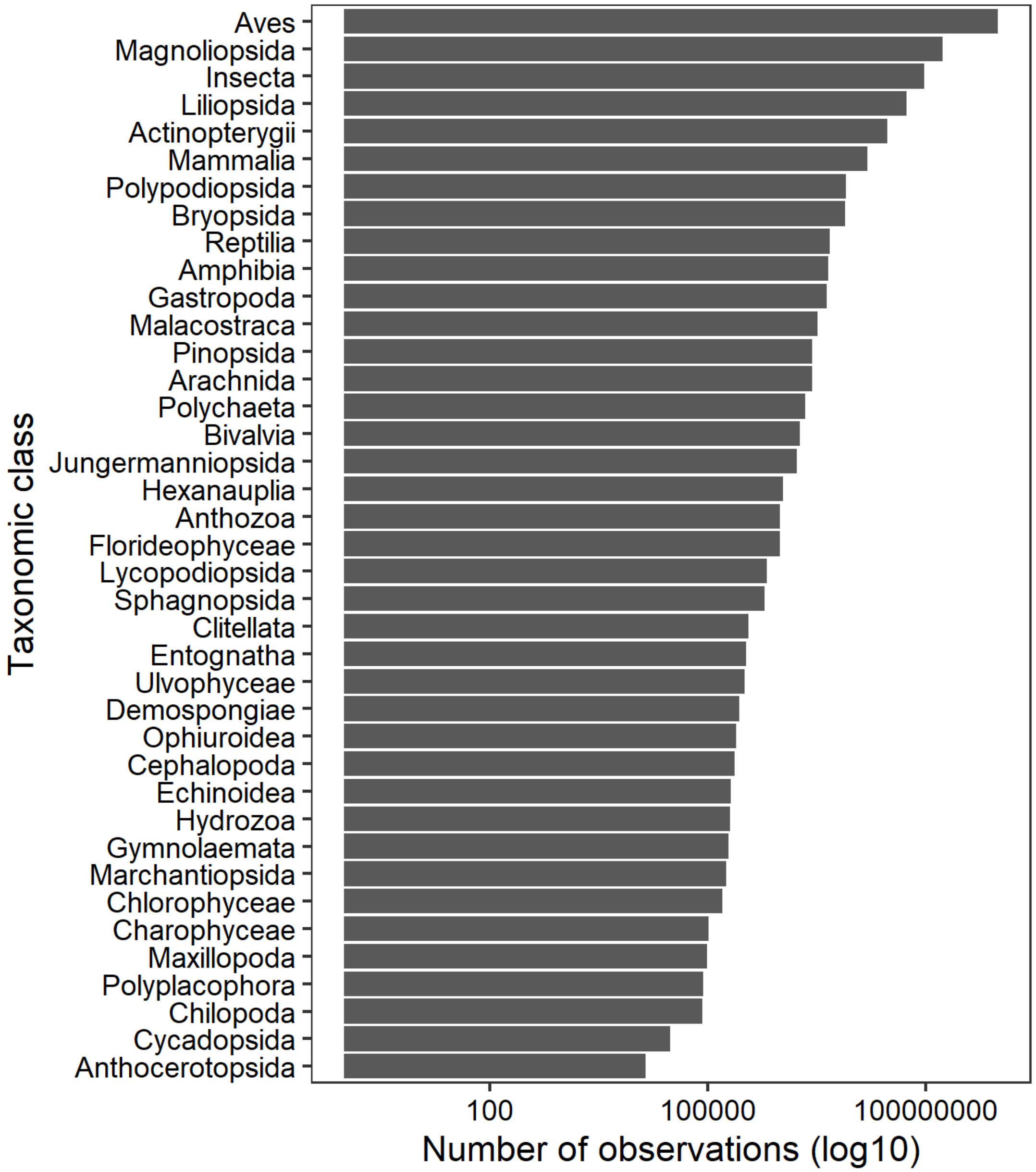

Fig. S1. The 39 classes included in our analyses, and the number of total observations used, present in GBIF from 1900-2019, for each class (on a log10-scale).

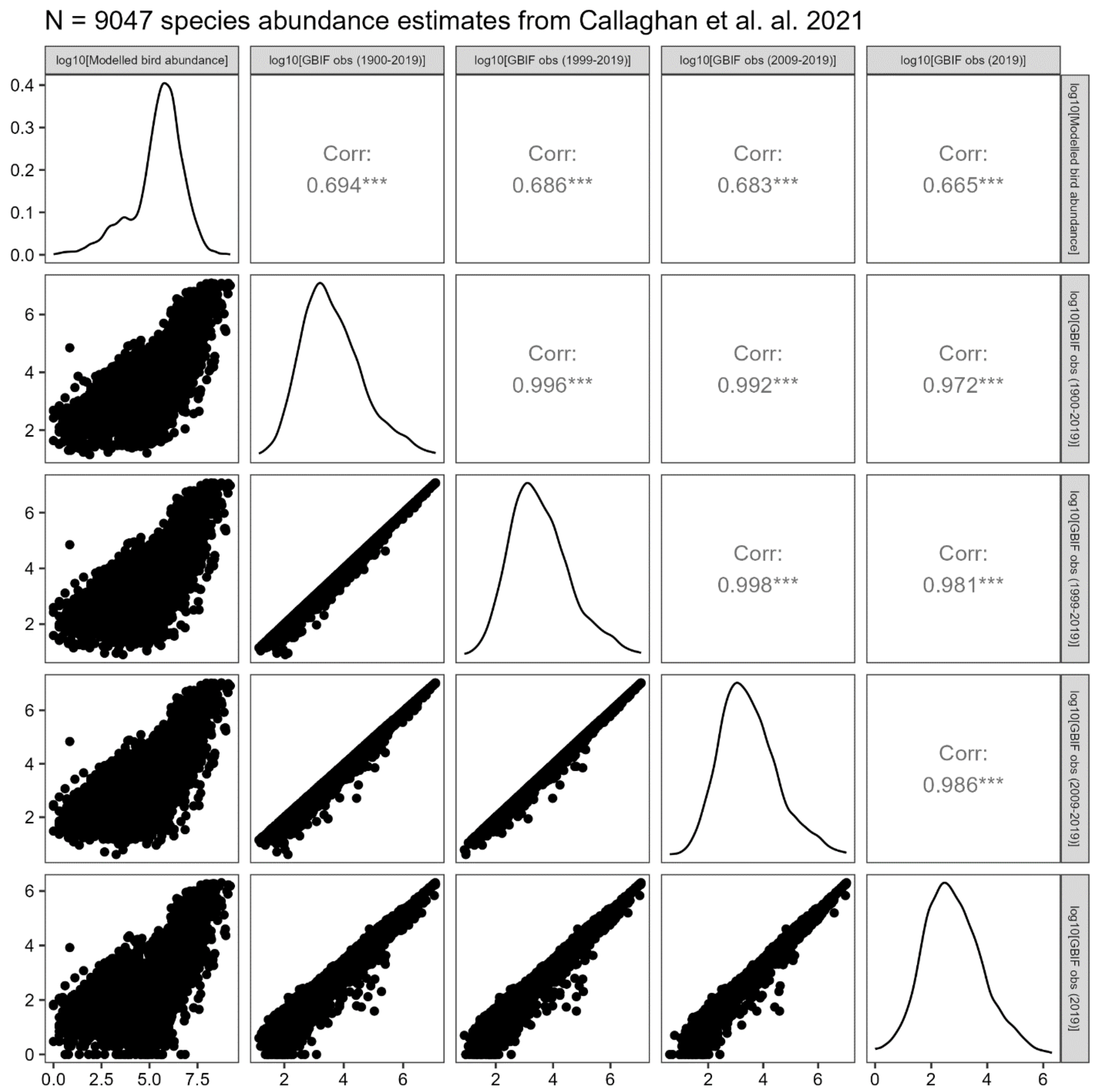

Fig. S2. The relationship between a global abundance estimate (in absolute numbers of individuals) from Callaghan et al. 2021 and the number of records in GBIF for 1900-2019 (cumulative), 1999-2019 (20 years cumulative), 2009-2019 (10 years cumulative), and just 2019 (individual year). There was a strong relationship for the 9,047 species assessed providing support to the notion of the number of GBIF occurrences serving as a proxy for the abundance of a species. All abundances are log10-transformed. The three asterisks (***) represent significance at the p<0.001 level from a Pearson two-sided correlation test. For a smaller subset of global abundance estimates, from a different source, see Fig. S3.

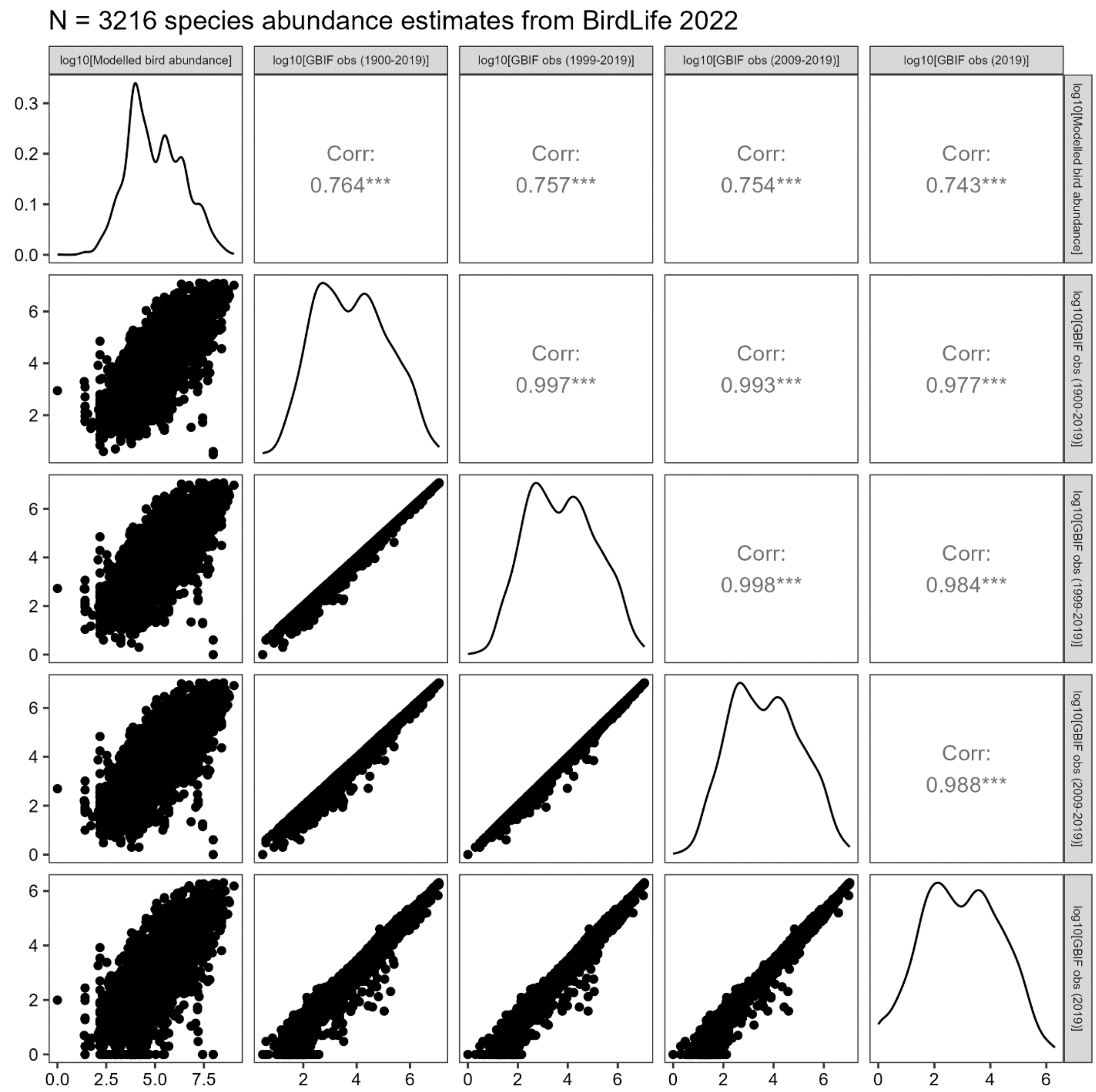

Fig. S3. The relationship between a global abundance estimate (in absolute numbers of individuals) from BirdLife International 2022 and the number of records in GBIF for 1900-2019 (cumulative), 1999-2019 (20 years cumulative), 2009-2019 (10 years cumulative), and just 2019 (individual year). There was a strong relationship for the 3216 species assessed providing support to the notion of the number of GBIF occurrences serving as a proxy for the abundance of a species. All abundances are log10-transformed. The three asterisks (***) represent significance at the $p<0.001$ level from a Pearson two-sided correlation test. For a larger subset of global abundance estimates, from a different source, see Fig. S2.

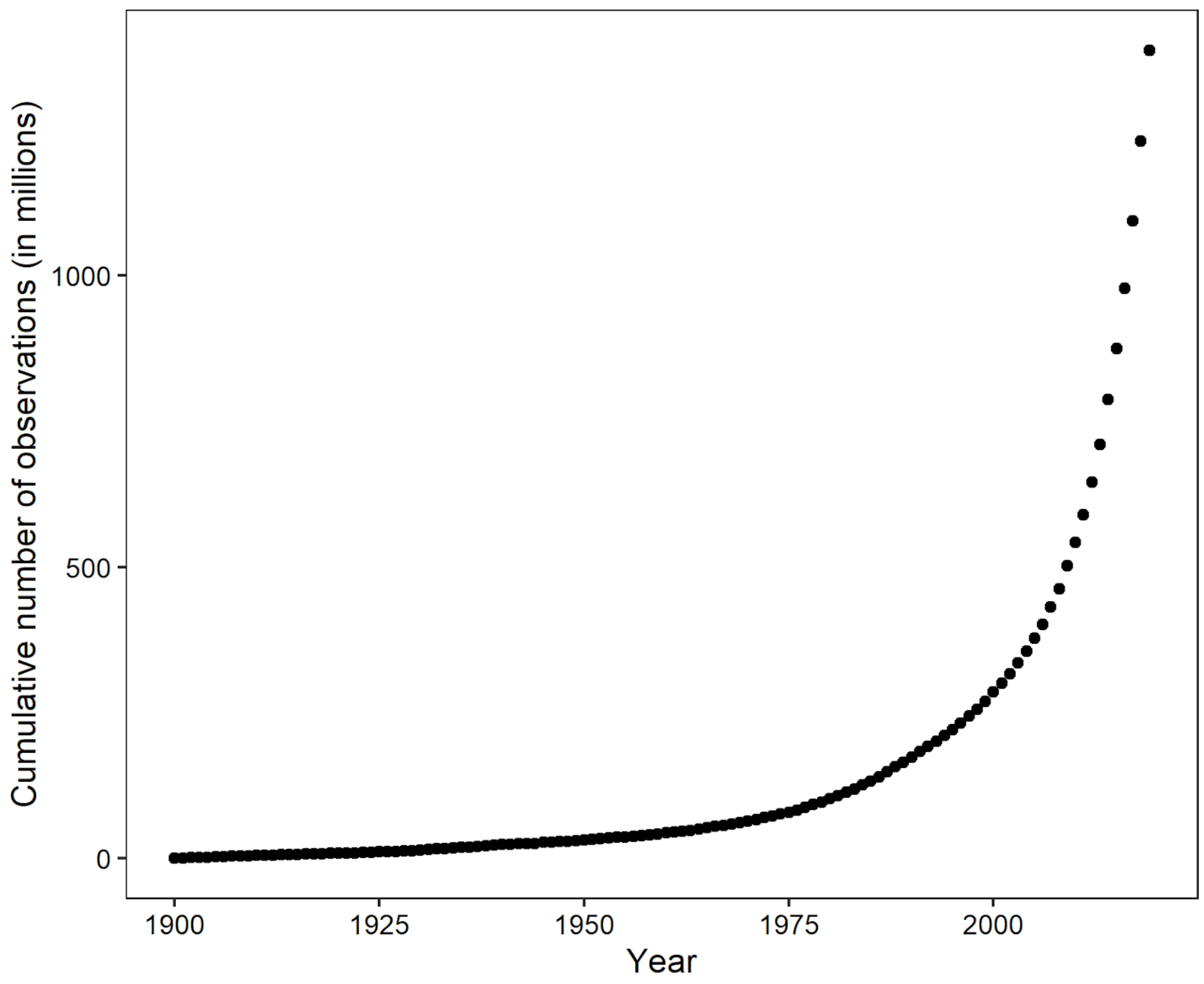

Fig. S4. The cumulative number of observations, across the 39 classes included in our analysis (Fig. S1), from 1900-2019.

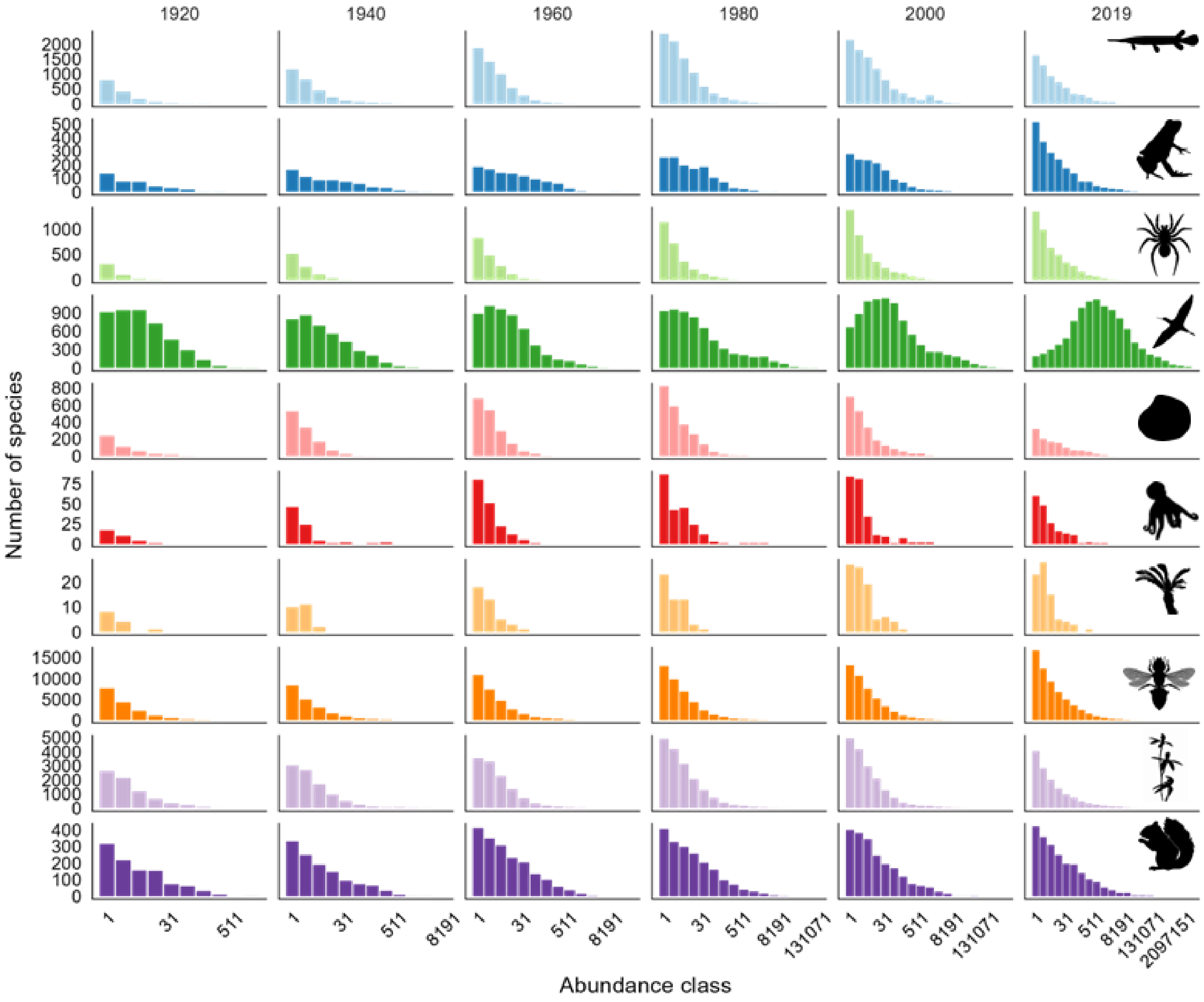

Fig. S5. The gSAD for each of 10 example classes, where each year is an individual year treated separately, in contrast to our analysis presented in the main text where each year represents a 20-year rolling window. Despite only being yearly, by the end of the time series, birds are still being fully sampled showing a log-left skew distribution. See animations for all 39 classes here.

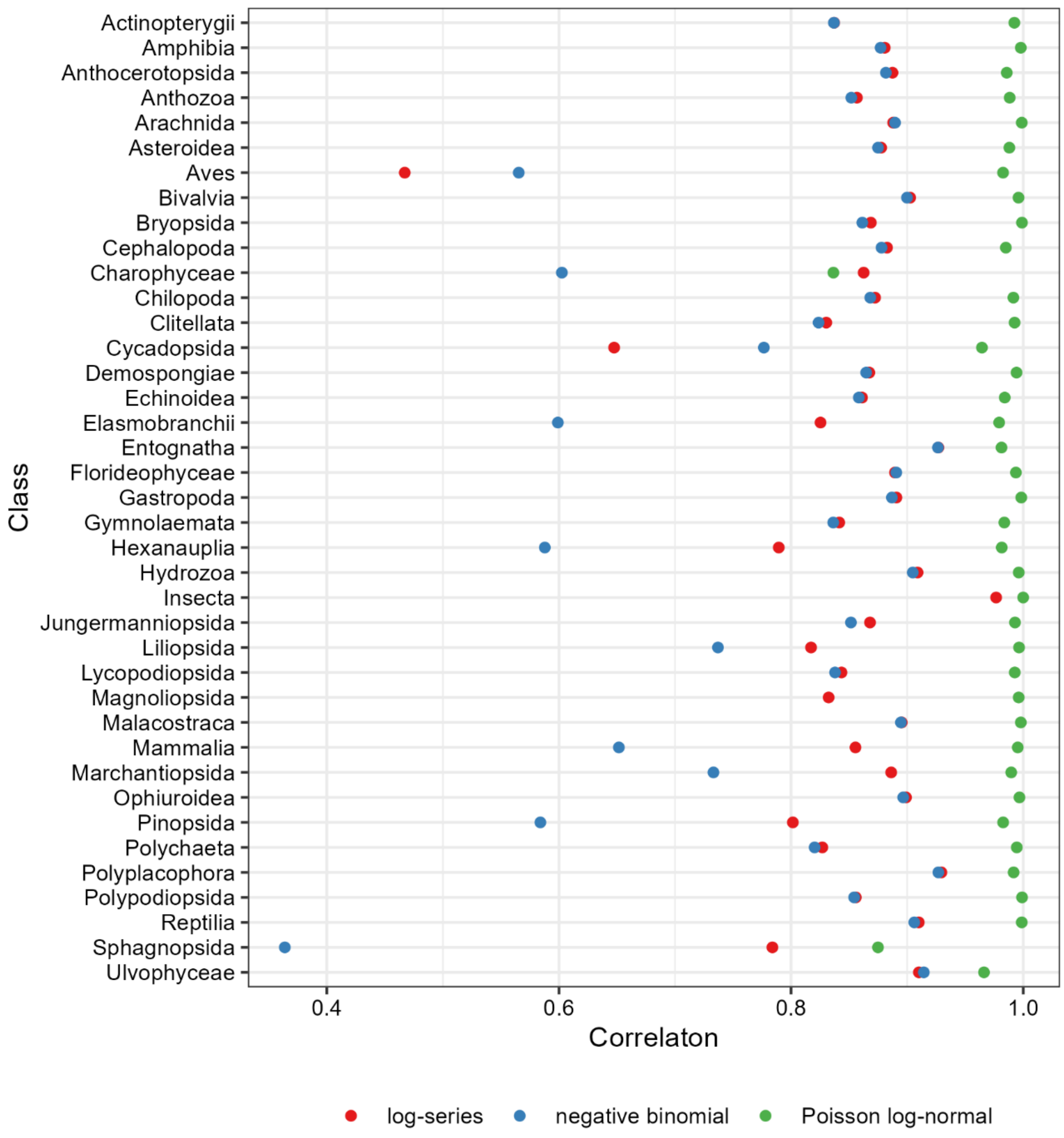

Fig. S6. The final 20-year rolling window correlation of predicted vs observed fitted values for each class in our analysis, showing that the Poisson log-normal had the best 'fit' for all but one (Charopyceae) class.

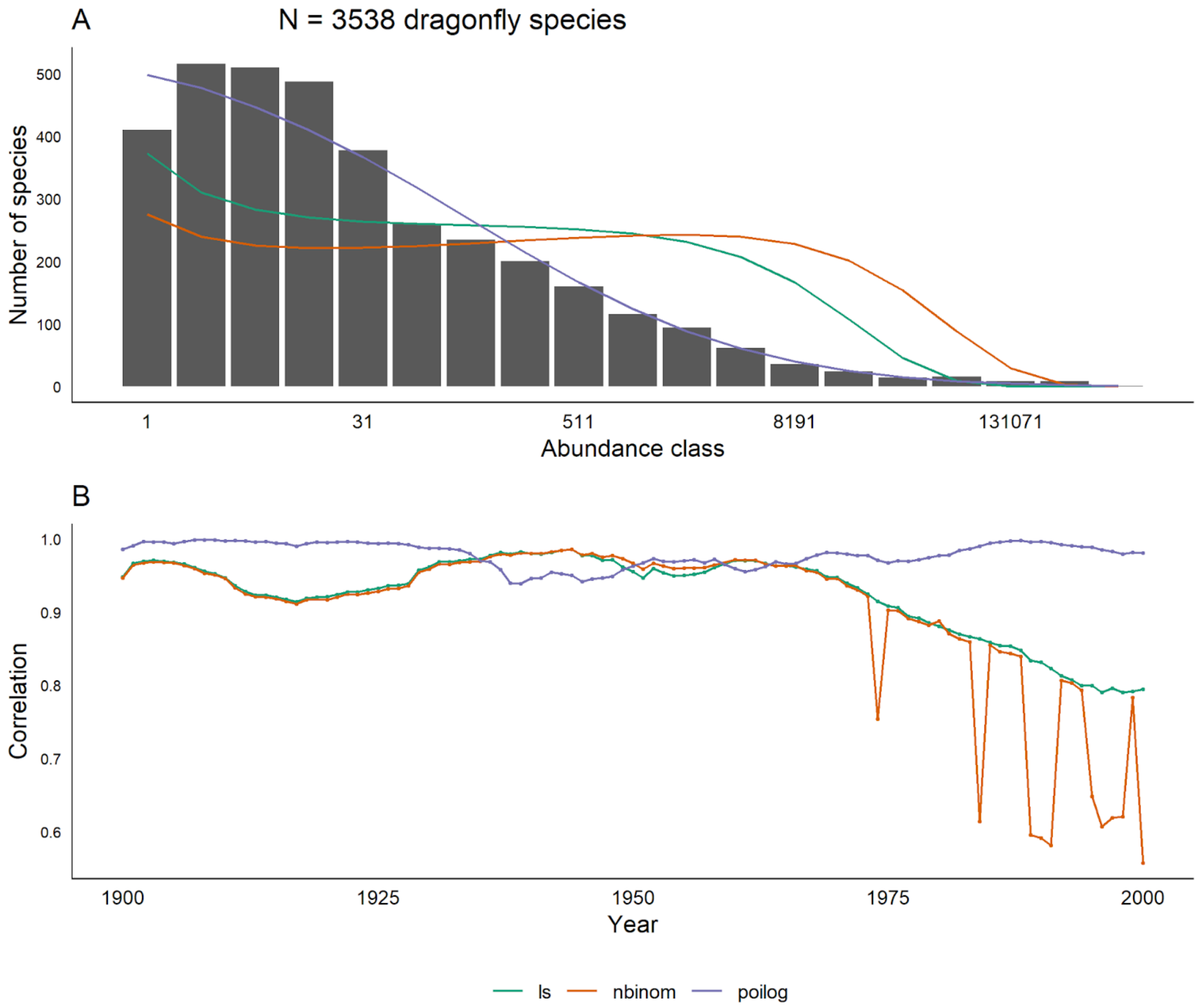

Fig. S7. An exploratory test of the gSAD for dragonfly species (N=3538) — a subset of Insecta as a whole. We used the methods described for the main analysis and tested the statistical fit of the log-series (ls; green line), negative binomial (nbinom; orange line), and Poisson log-normal (poilog; purple line) distributions. The aggregation method shown is the 20-year rolling window.

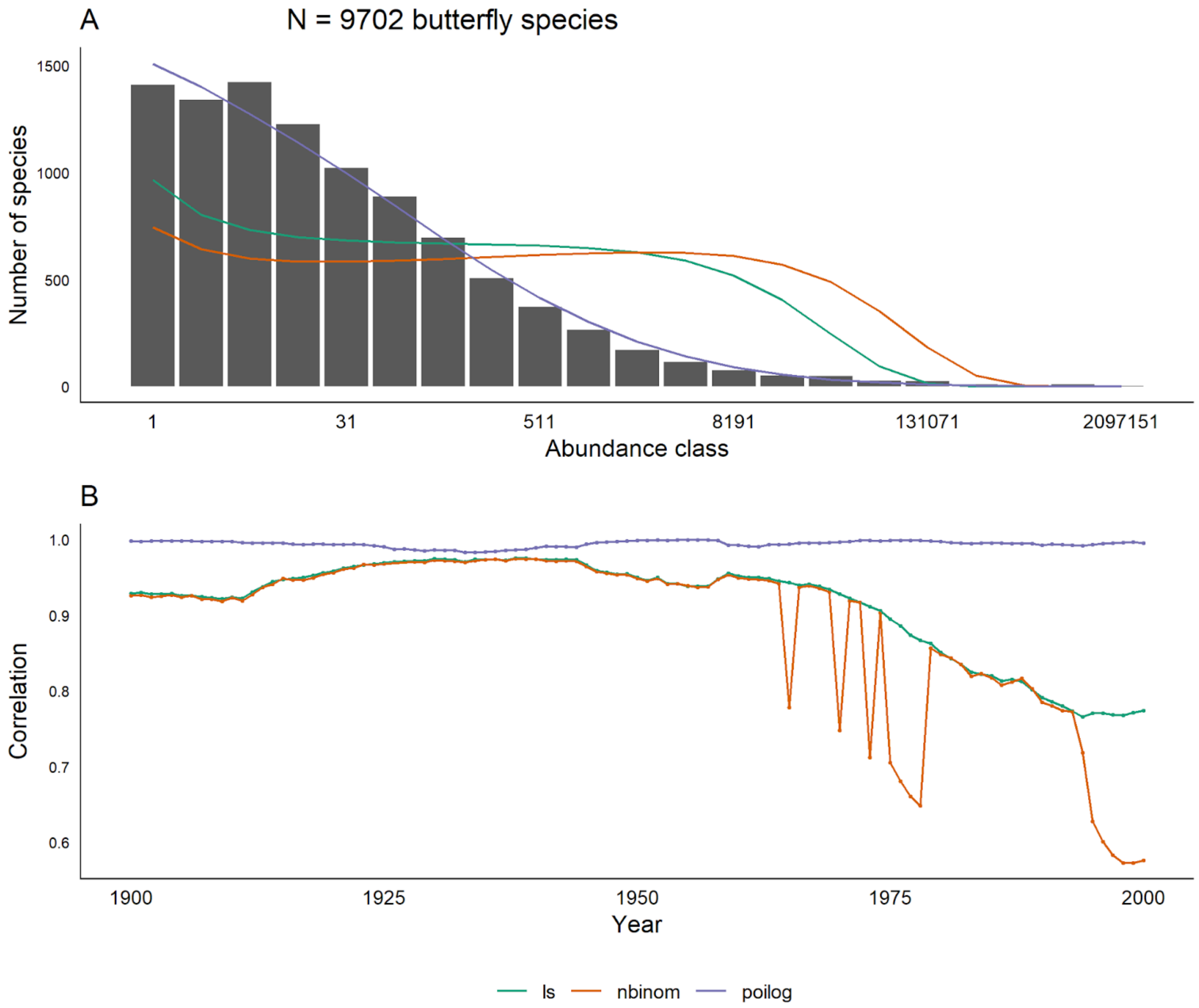

Fig. S8. An exploratory test of the gSAD for butterfly species (N=9702) — a subset of Insecta as a whole. We used the methods described for the main analysis and tested the statistical fit of the log-series (ls; green line), negative binomial (nbinom; orange line), and Poisson log-normal (poilog; purple line) distributions. The aggregation method shown is the 20-year rolling window.

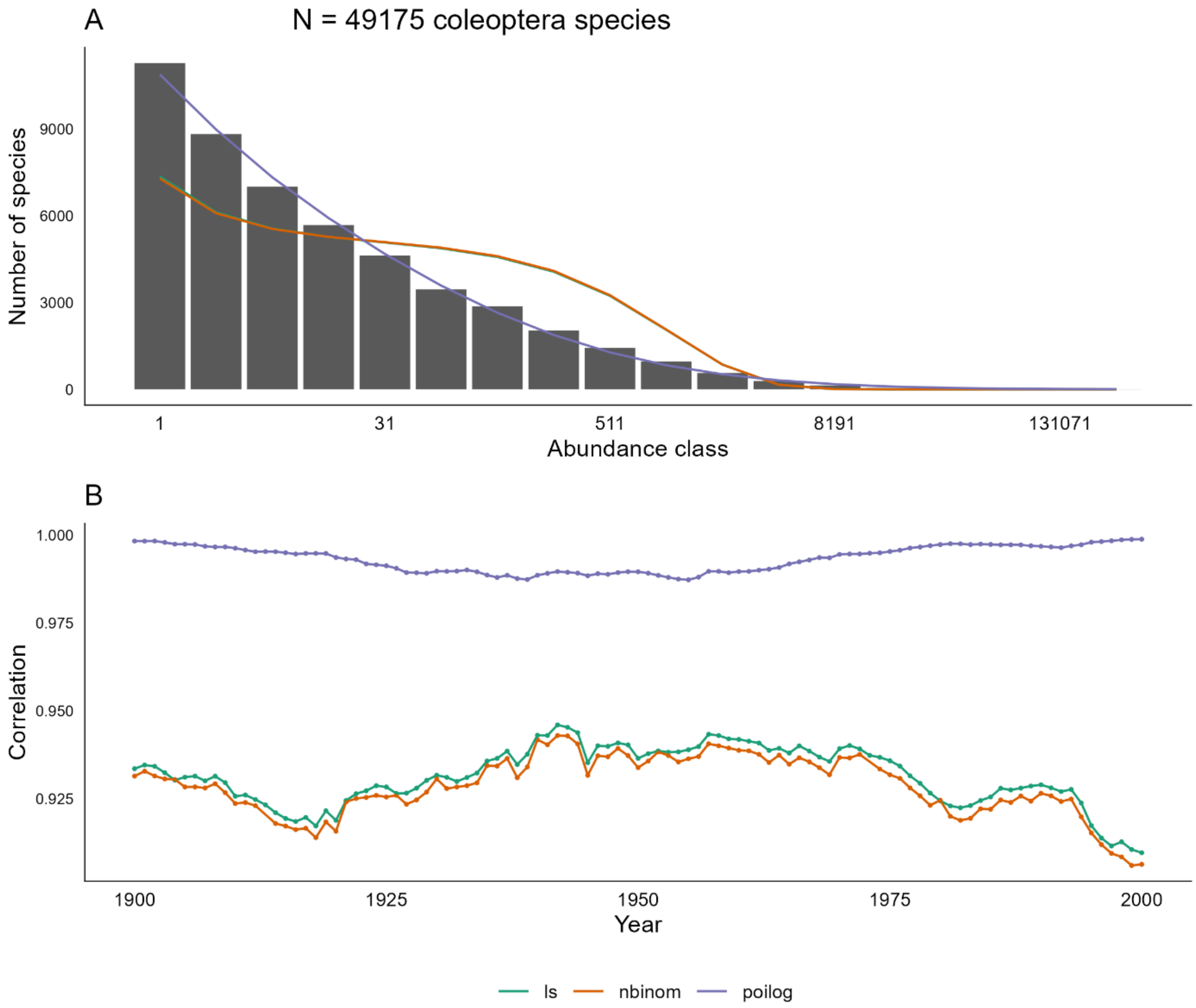

Fig. S9. An exploratory test of the gSAD for Coleoptera species (N=49175) — a subset of Insecta as a whole. We used the methods described for the main analysis and tested the statistical fit of the log-series (ls; green line), negative binomial (nbinom; orange line), and Poisson log-normal (poilog; purple line) distributions. The aggregation method shown is the 20-year rolling window.

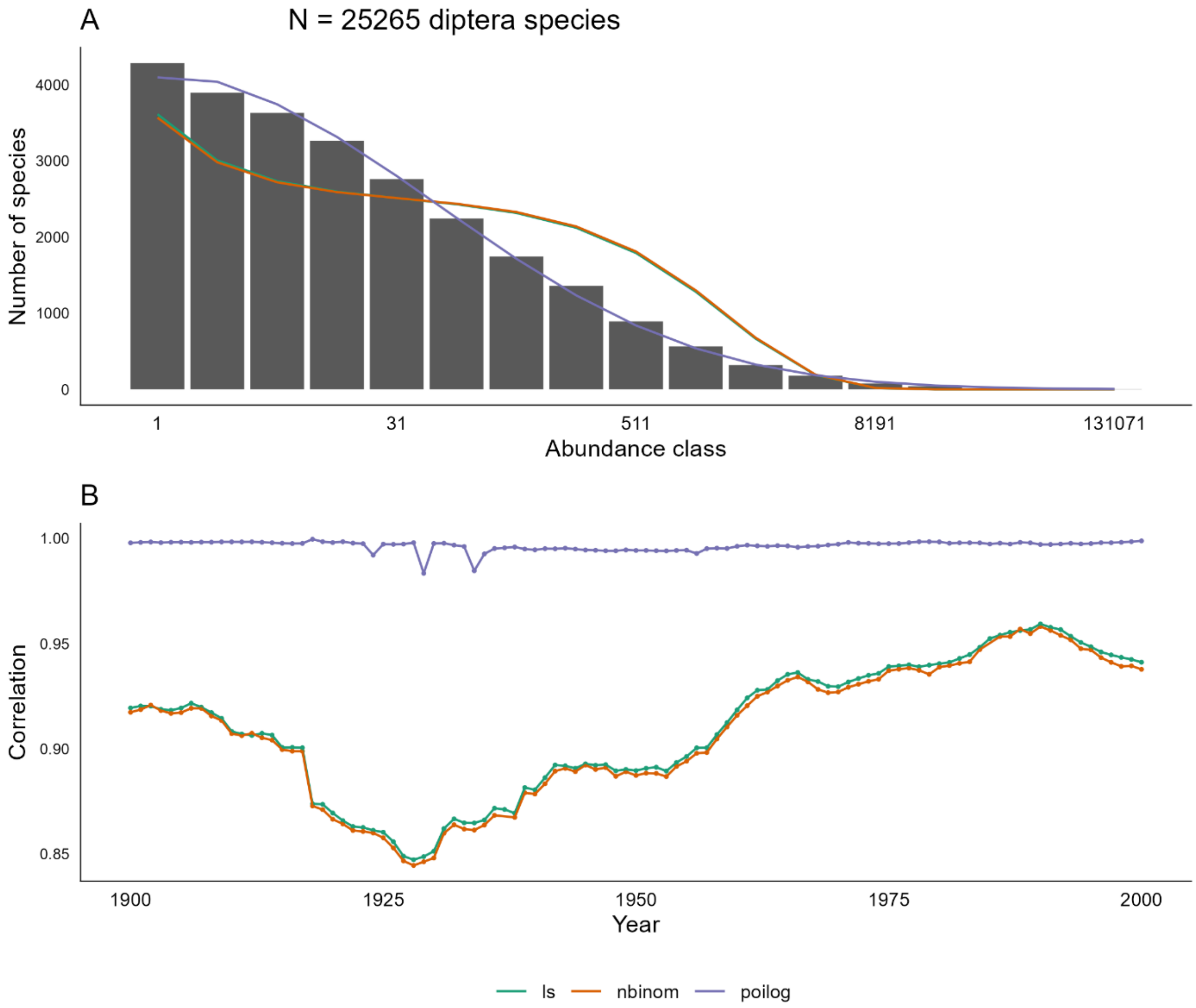

Fig. S10. An exploratory test of the gSAD for Diptera species (N=25265) — a subset of Insecta as a whole. We used the methods described for the main analysis and tested the statistical fit of the log-series (ls; green line), negative binomial (nbinom; orange line), and Poisson log-normal (poilog; purple line) distributions. The aggregation method shown is the 20-year rolling window.

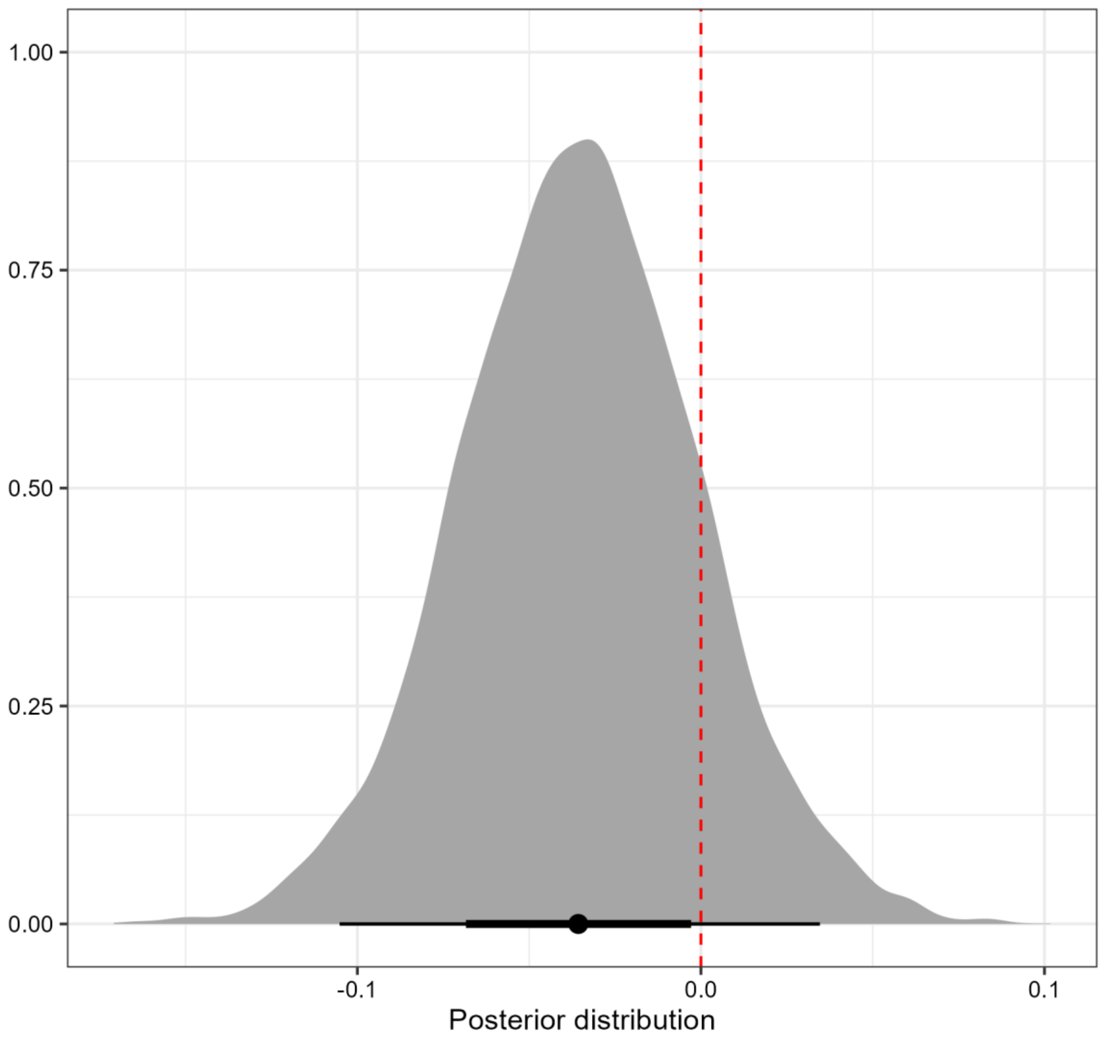

Fig. S11. A posterior distribution of a Bayesian model fit for the relationship between the percentage of the gSAD uncovered and the total observed species richness in a taxonomic class. Models were fit using n=39 independent classes as replicates. The more observed species in a class, the further away from the gSAD was of being fully sampled. The bars represent the posterior median (the dot), 66%, and 95% quantile intervals.

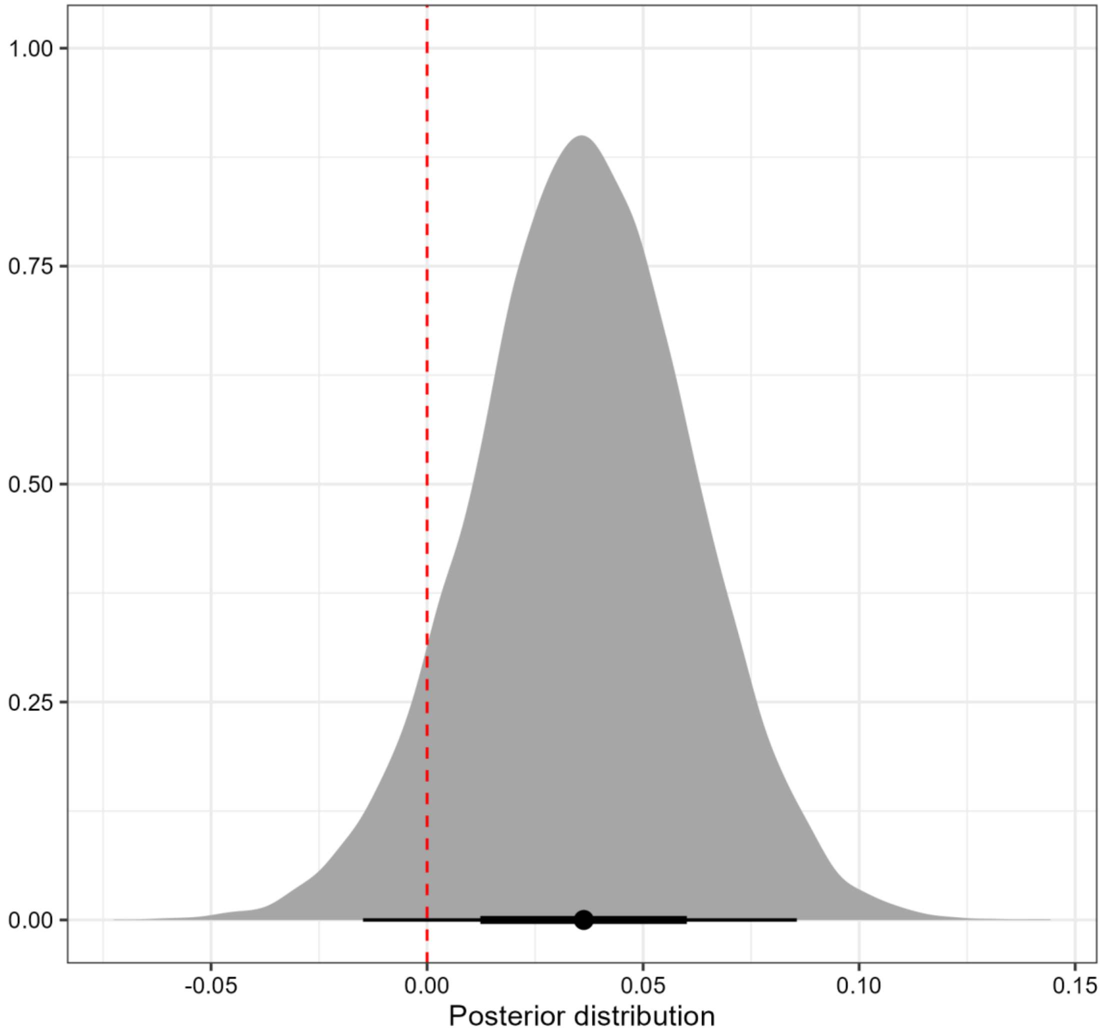

Fig. S12. A posterior distribution of a Bayesian model fit for the relationship between the percentage of the gSAD uncovered and the total number of observations in a taxonomic class. Models were fit using n=39 independent classes as replicates. The more observations in a class, the closer the gSAD was of being fully sampled. The bars represent the posterior median (the dot), 66%, and 95% quantile intervals.

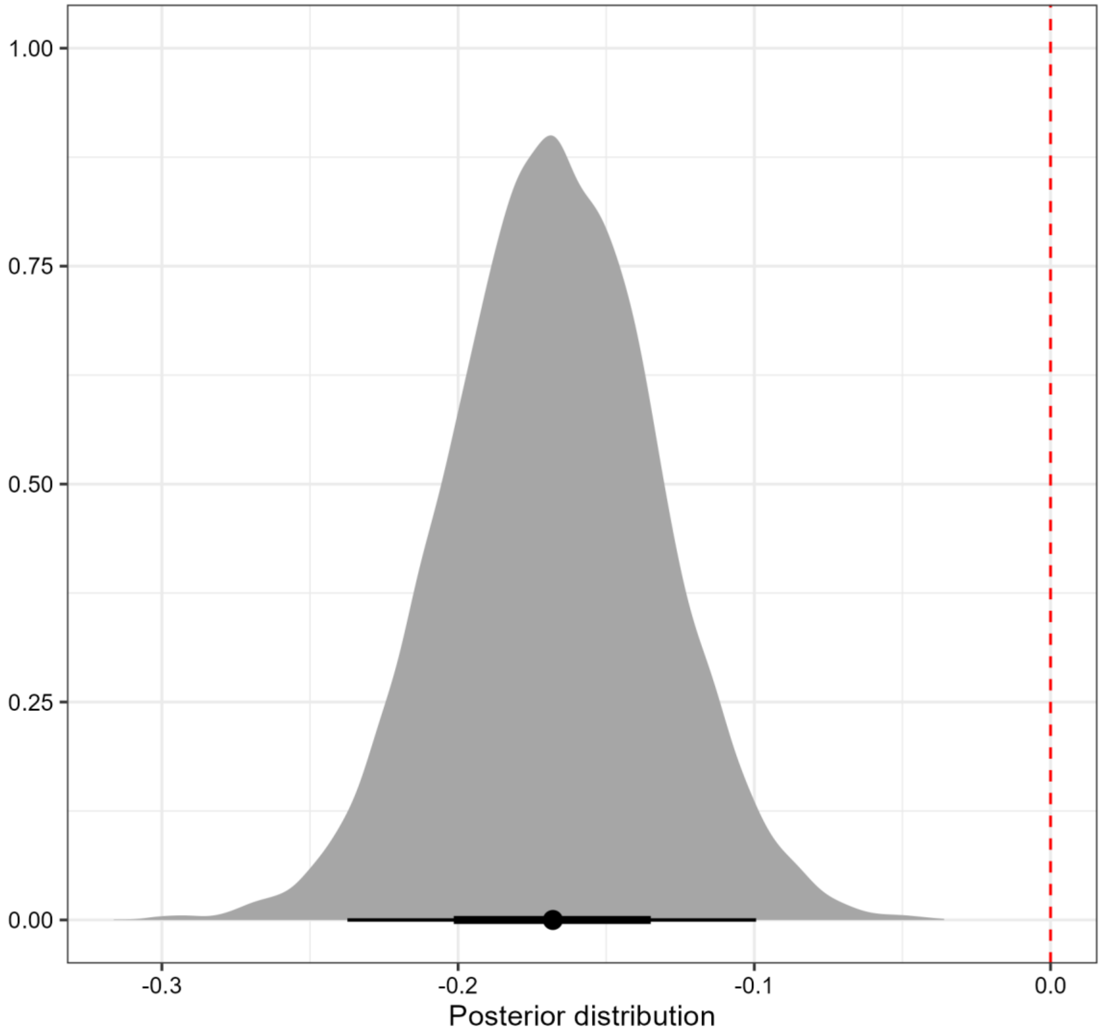

Fig. S13. A posterior distribution of a Bayesian model fit for the relationship between the percentage of the gSAD uncovered and the total observed species richness divided by the total number of observations. Models were fit using n=39 independent classes as replicates. The lower the proportional sampling in a class (i.e., the more observations per species) the closer the gSAD was of being fully sampled. The bars represent the posterior median (the dot), 66%, and 95% quantile intervals.

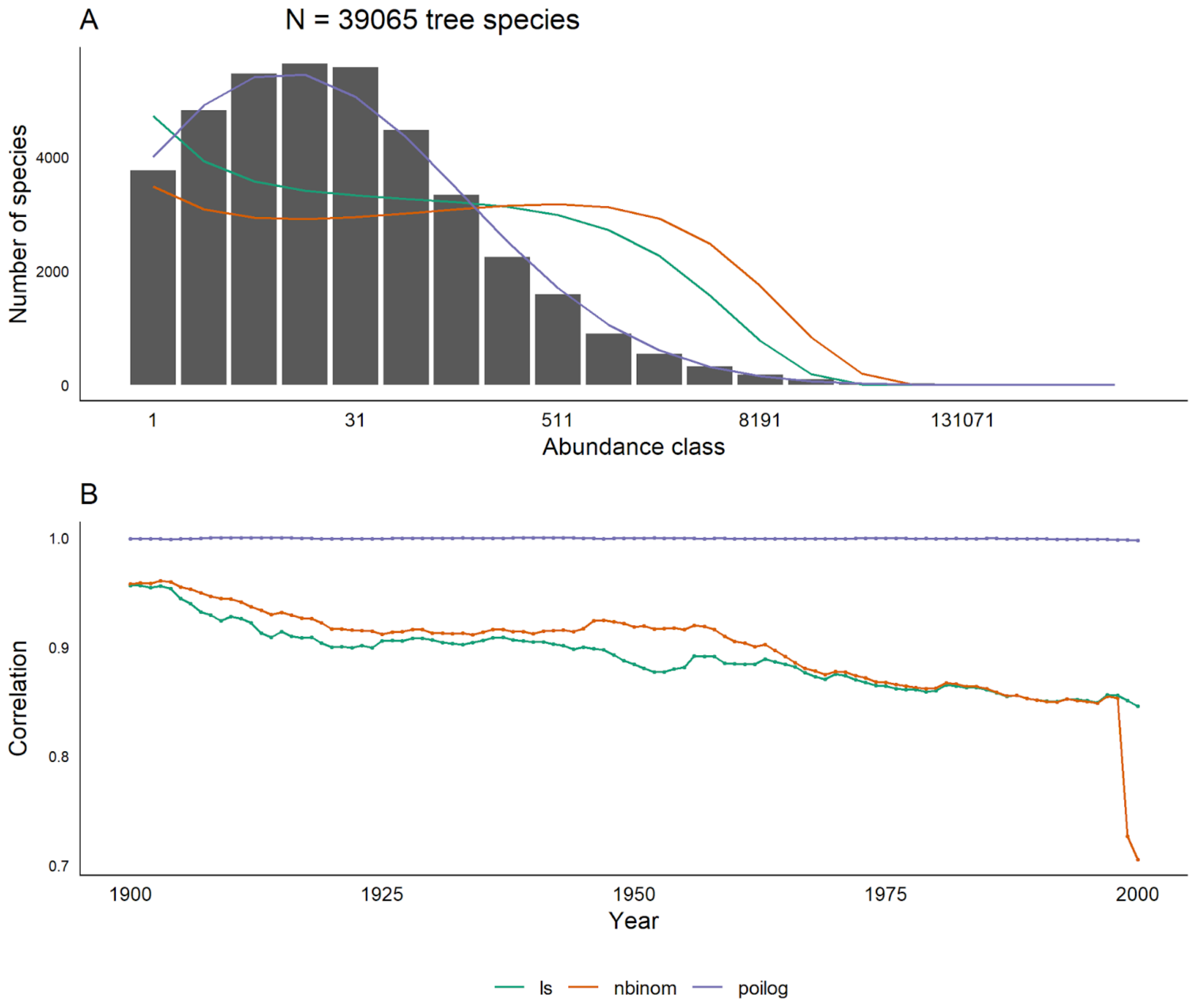

Fig. S14. The global species abundance distribution for 39,065 tree species, as defined by the Botanic Gardens Conservation International global tree list (*36*), showing that the Poisson log-normal is the superior fit both in the last 20-year rolling window (A) and through time (B). The aggregation method shown is the 20-year rolling window.

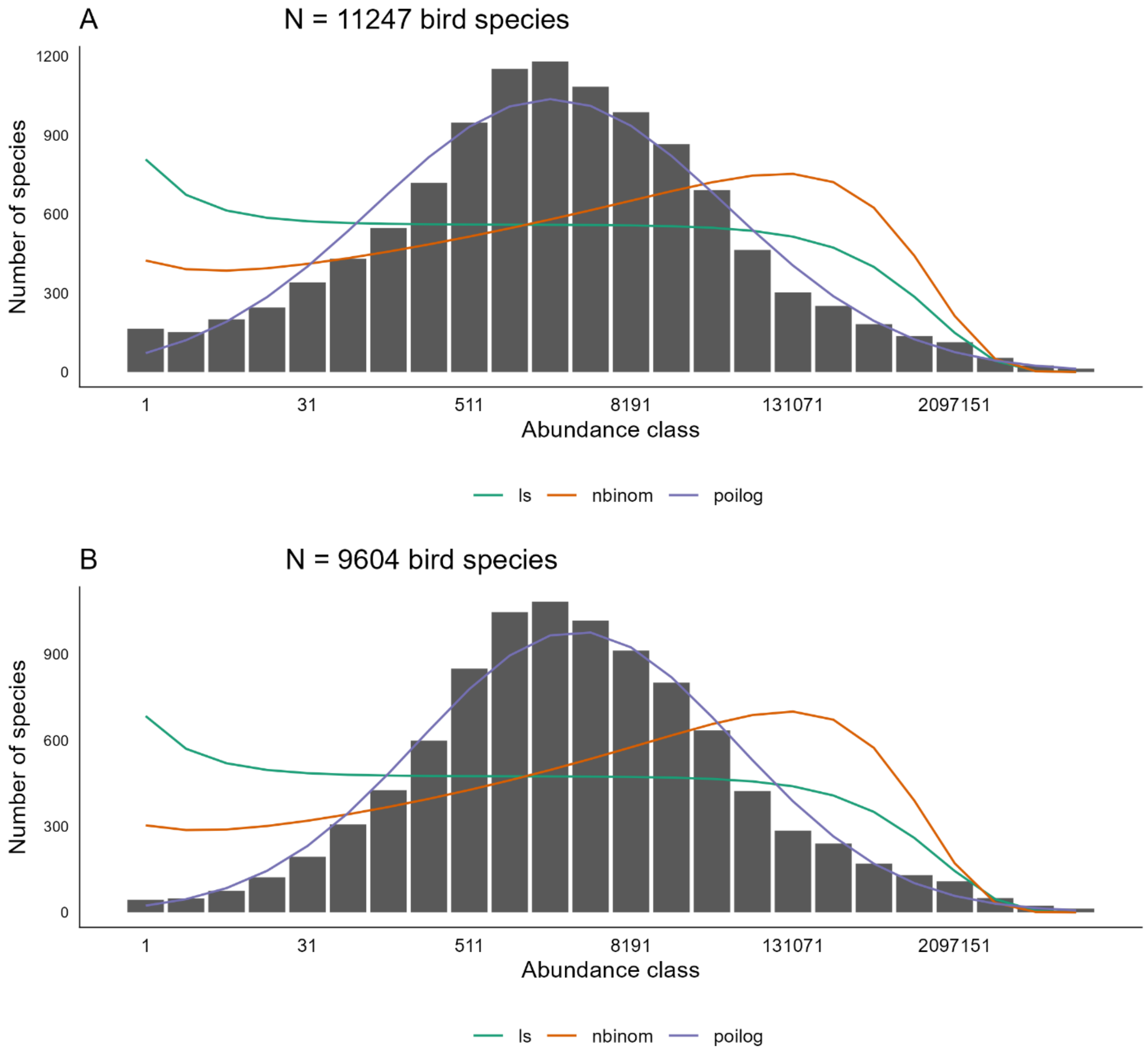

Fig. S15. Exploratory analysis for the class Aves, showing that when trimming the GBIF species 'taxonomy' (A) to only those species included in an official bird taxonomy checklist (B) the same qualitative and quantitative results are returned. For this reason, because we were assessing 39 different classes that would present a computation bottleneck to resolve taxonomies for each class, we use the GBIF 'taxonomy' throughout our main results. The aggregation method shown is the 20-year rolling window.

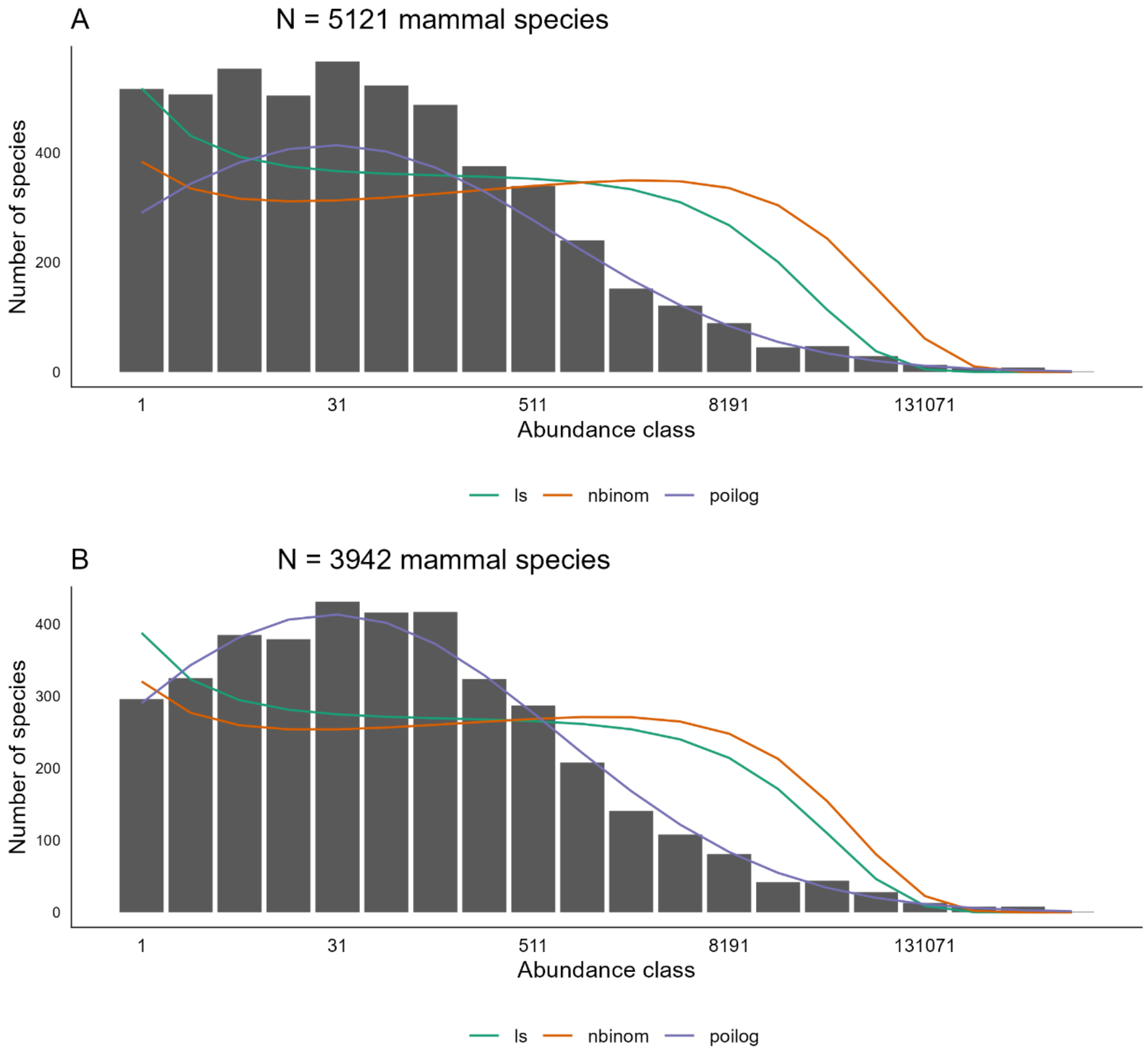

Fig. S16. Exploratory analysis for the class Mammalia, showing that when trimming the GBIF species 'taxonomy' (A) to only those species included in an official mammal taxonomy checklist (B) the same qualitative and quantitative results are returned. For this reason, because we were assessing 39 different classes that would present a computation bottleneck to resolve taxonomies for each class, we use the GBIF 'taxonomy' throughout our main results. The aggregation method shown is the 20-year rolling window.

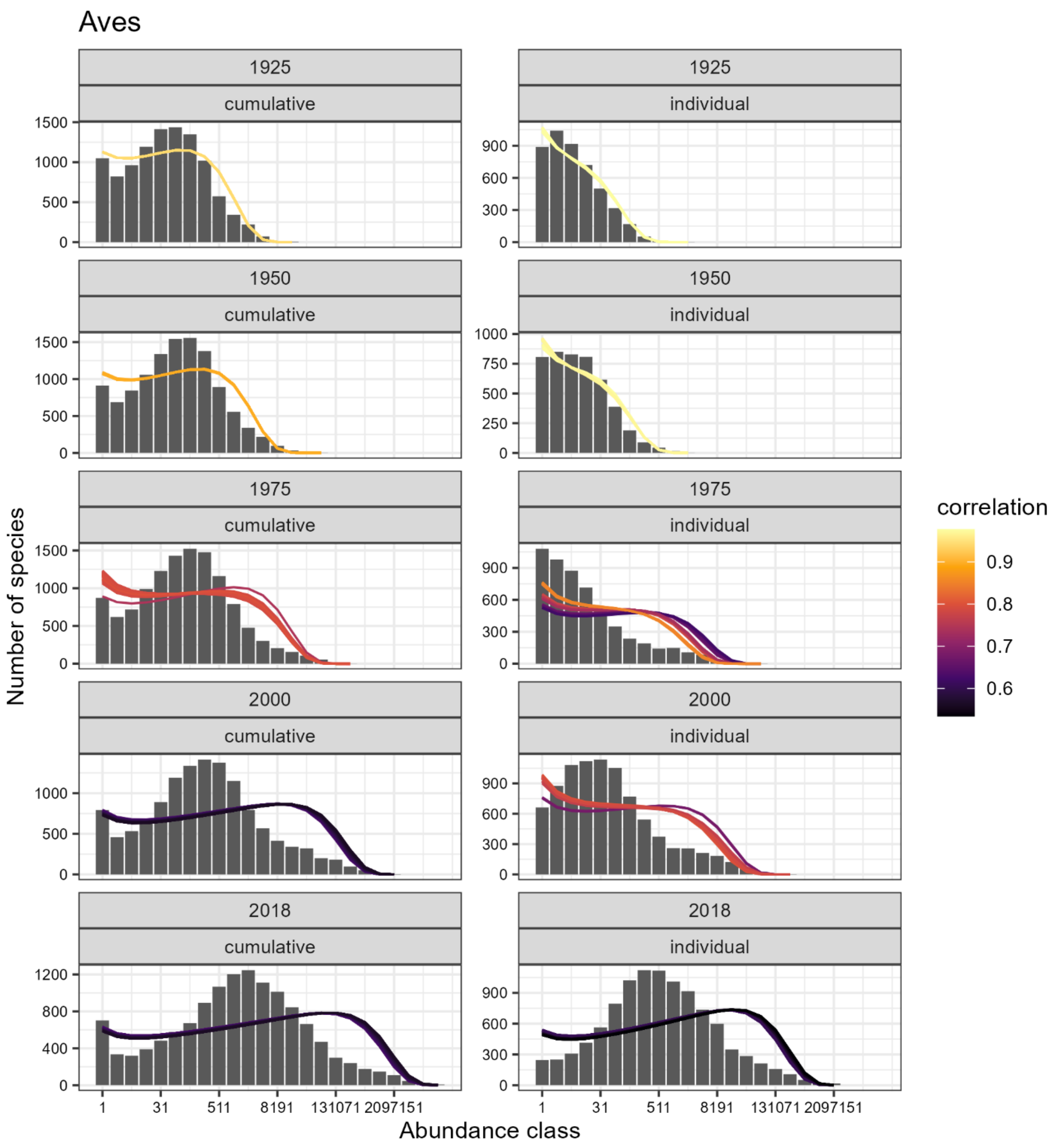

Fig. S17. Exploratory analyses showing the robustness of the negative binomial fitting procedure by adjusting the starting parameter values, mu, and r. The results here are presented for Aves, and illustrate that regardless of starting parameter values (each line in a panel), the negative binomial produced a poor fit (color of lines is equal to the correlation value between observed and fitted distribution).

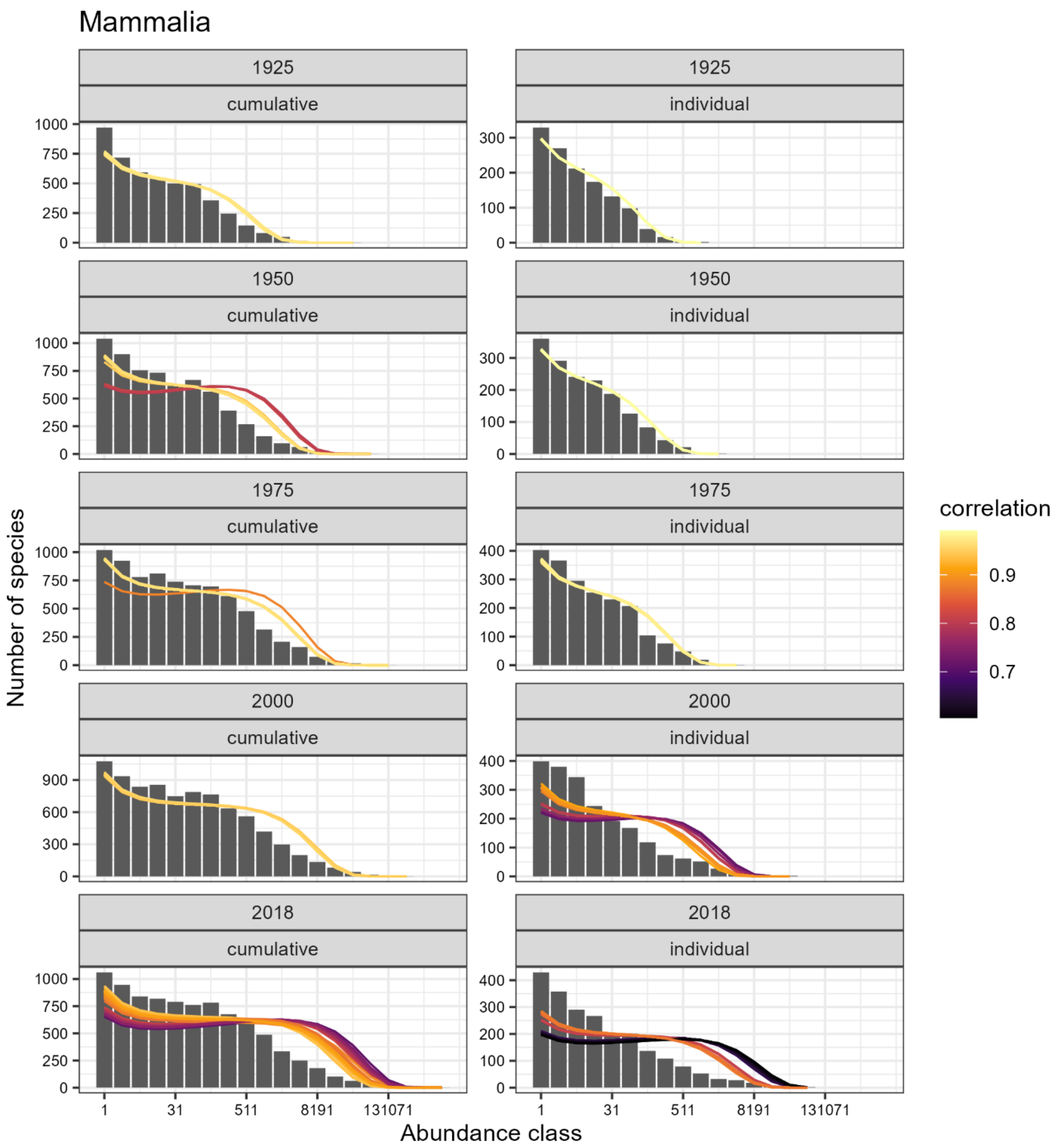

Fig. S18. Exploratory analyses showing the robustness of the negative binomial fitting procedure by adjusting the starting parameter values, mu, and r. The results here are presented for Mammalia, and illustrate that regardless of starting parameter values (each line in a panel), the negative binomial produced a poor fit (color of lines is equal to the correlation value between observed and fitted distribution).

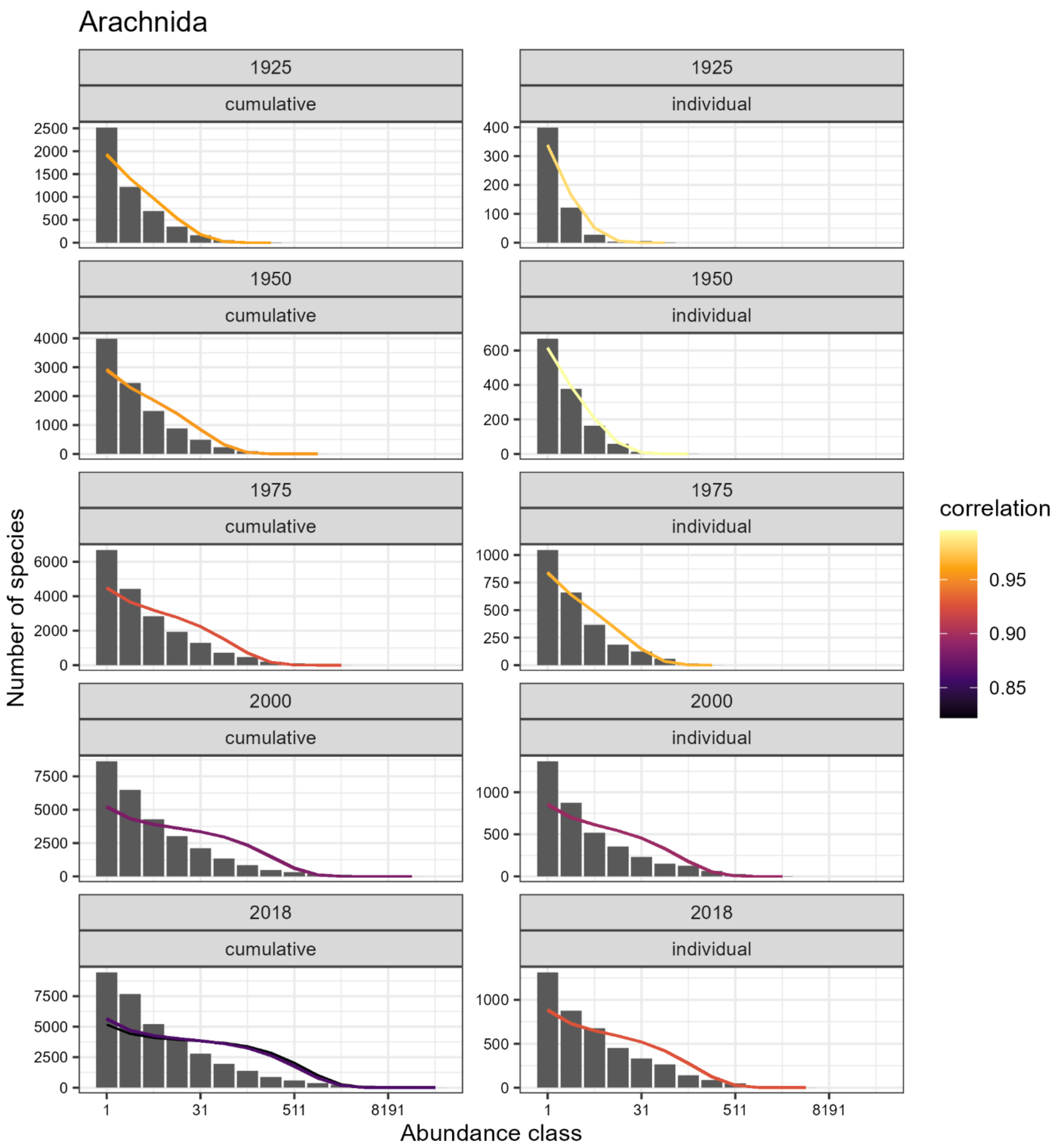

Fig. S19. Exploratory analyses showing the robustness of the negative binomial fitting procedure by adjusting the starting parameter values, mu, and r. The results here are presented for Arachnida, and illustrate that regardless of starting parameter values (each line in a panel), the negative binomial produced a poor fit (color of lines is equal to the correlation value).

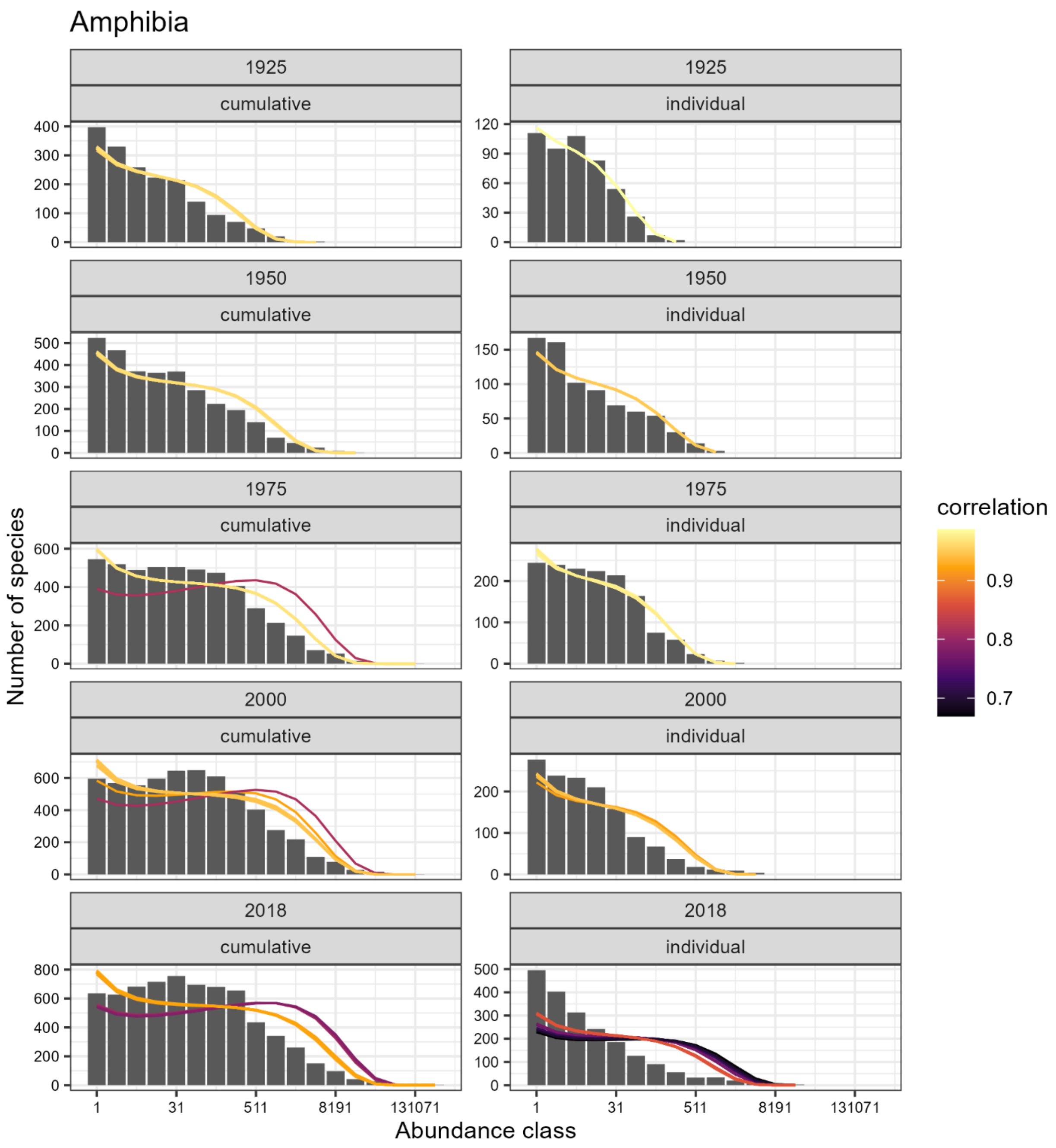

Fig. S20. Exploratory analyses showing the robustness of the negative binomial fitting procedure by adjusting the starting parameter values, mu, and r. The results here are presented for Amphibia, and illustrate that regardless of starting parameter values (each line in a panel), the negative binomial produced a poor fit (color of lines is equal to the correlation value).

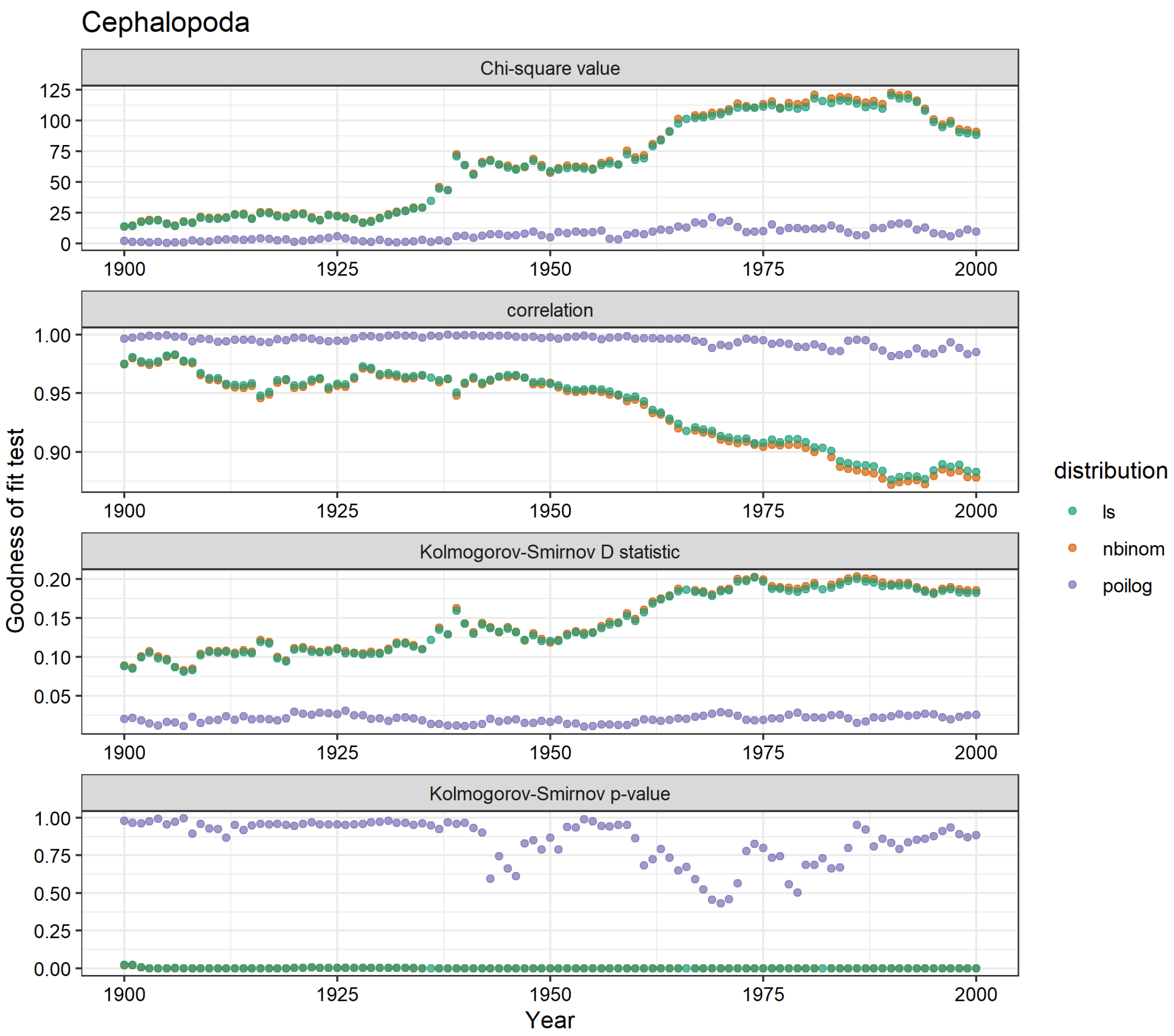

Fig. S21. Exploratory analyses illustrating that different measures of goodness of fit show similar trends in our understanding of the gSAD through time, where Poisson log-normal gains importance and becomes the statistically best fit by the end of the time series. Illustrated here is the fit for Cephalopoda. The Chi-sqaure test was two-sided.

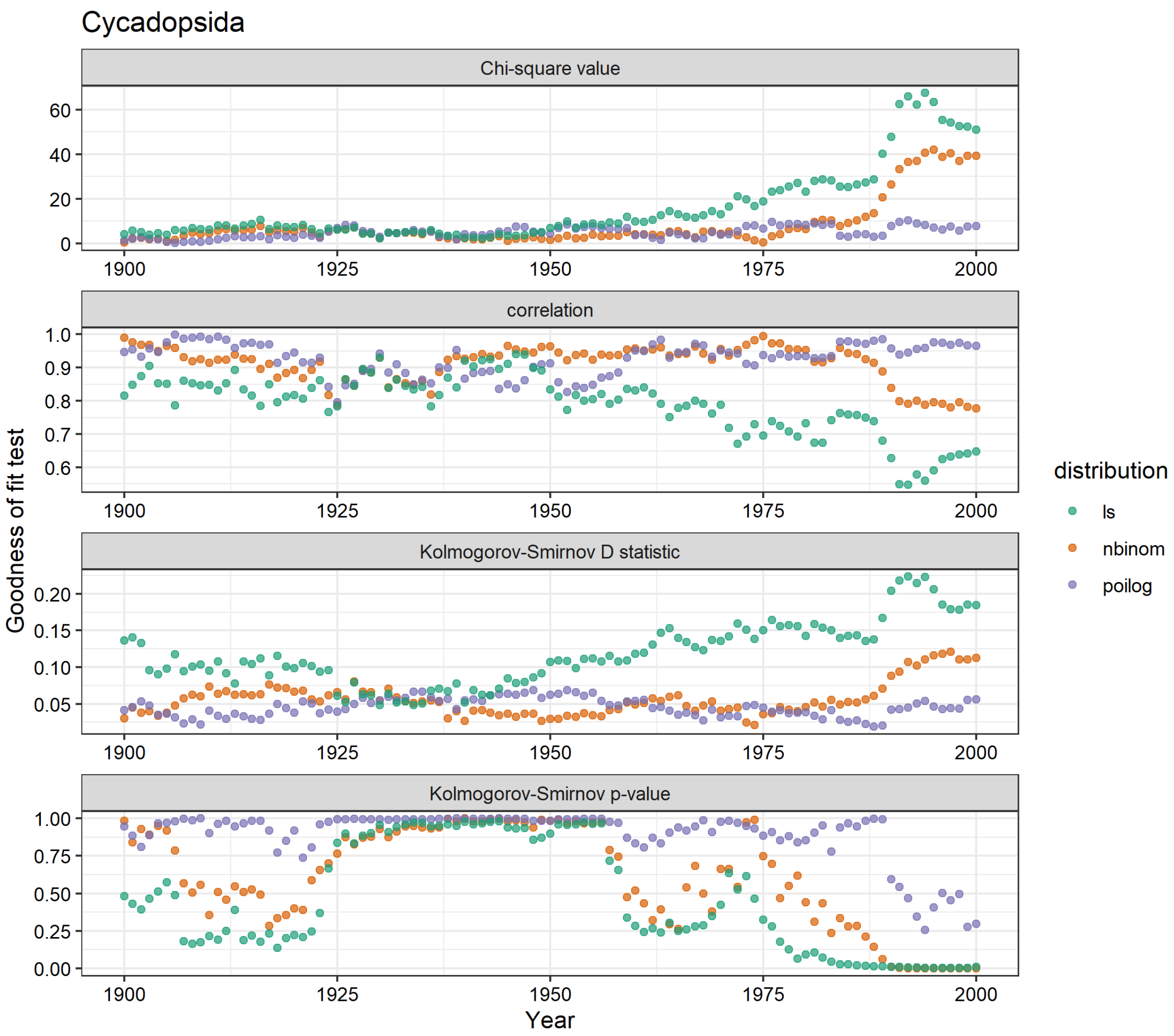

Fig. S22. Exploratory analyses illustrating that different measures of goodness of fit show similar trends in our understanding of the gSAD through time, where Poisson log-normal gains importance and becomes the statistically best fit by the end of the time series. Illustrated here is the fit for Cycadopsida. The Chi-sqaure test was two-sided.

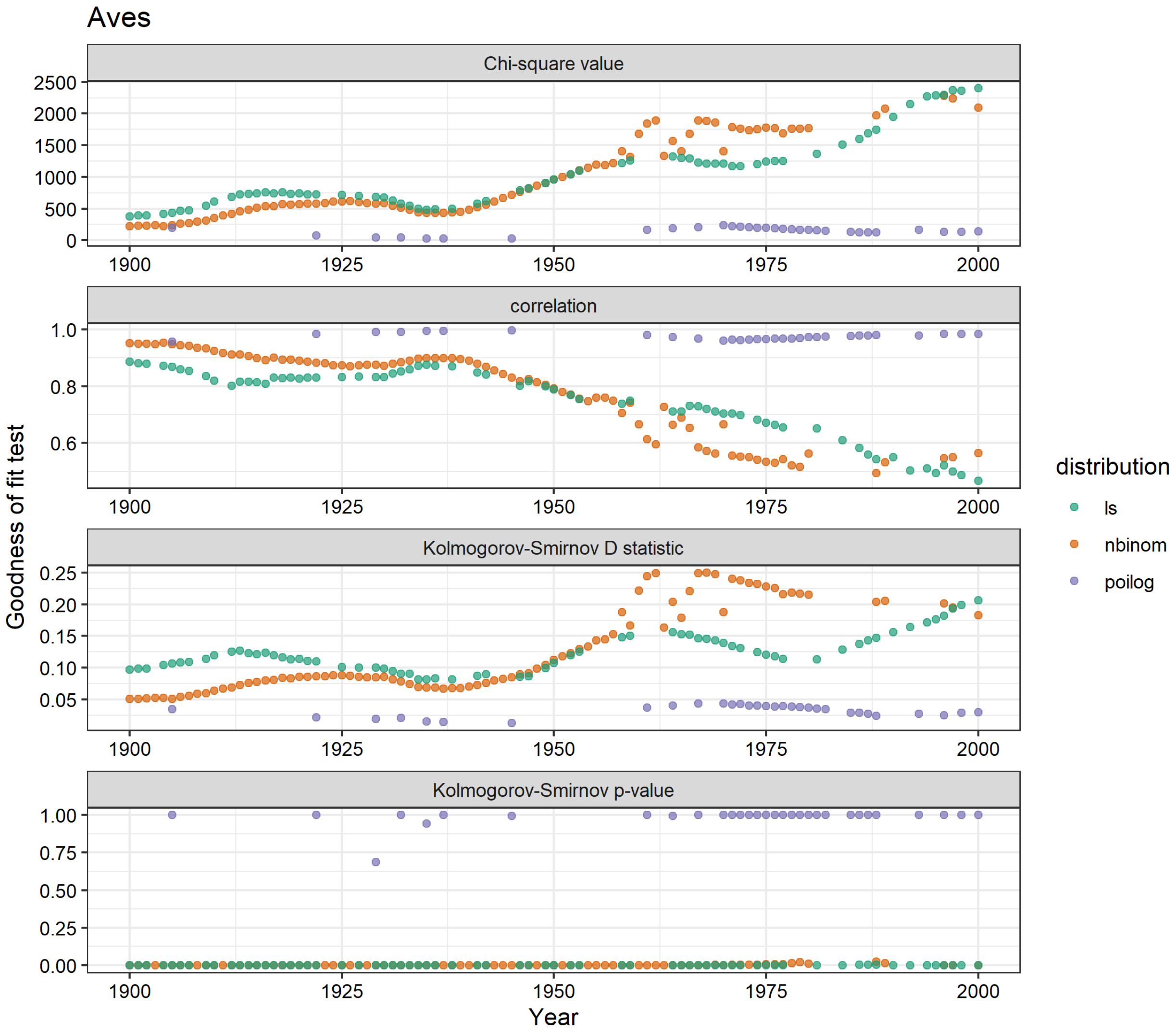

Fig. S23. Exploratory analyses illustrating that different measures of goodness of fit show similar trends in our understanding of the gSAD through time, where Poisson log-normal gains importance and becomes the statistically best fit by the end of the time series. Illustrated here is the fit for Aves. The Chi-sqaure test was two-sided.

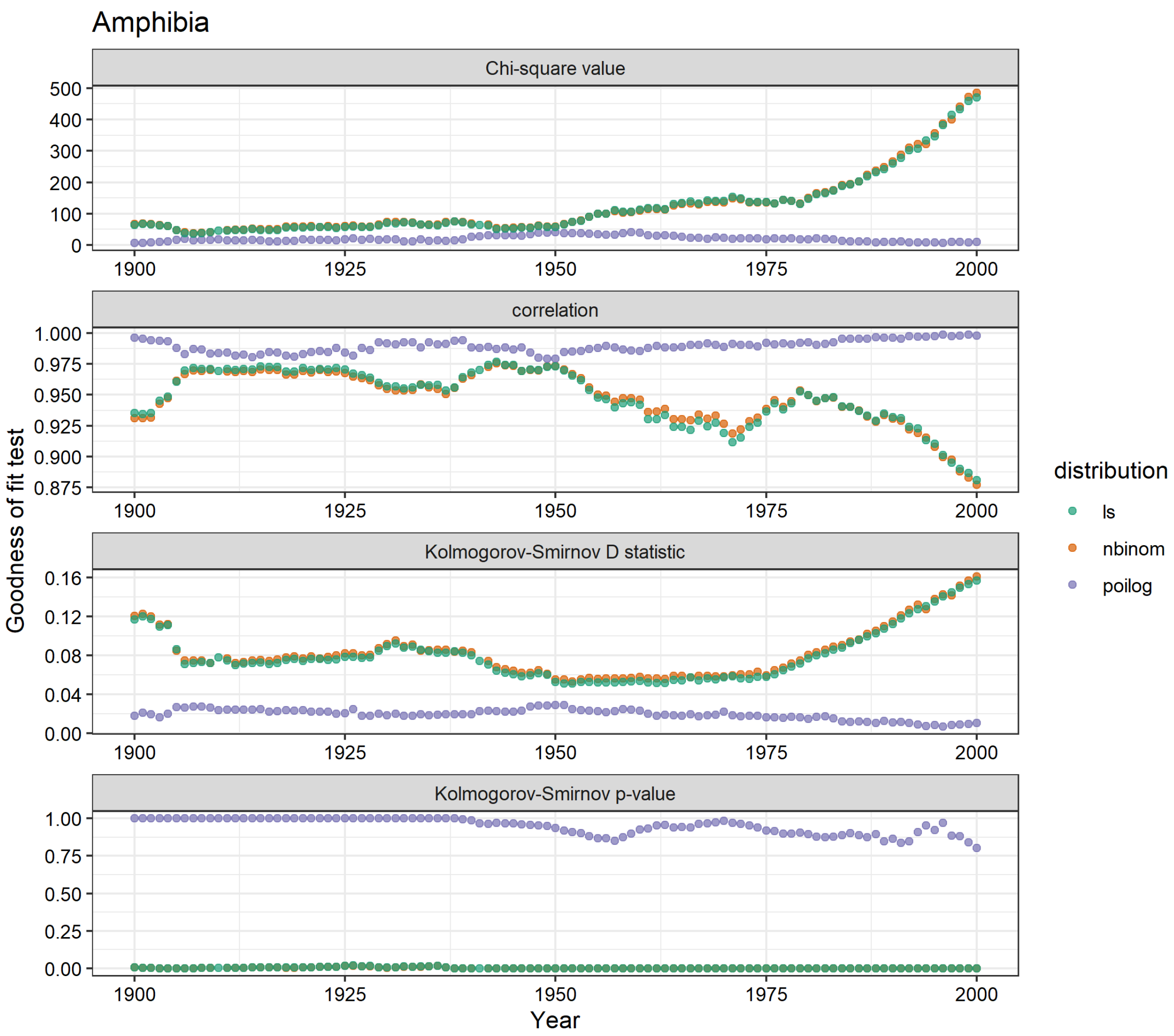

Fig. S24. Exploratory analyses illustrating that different measures of goodness of fit show similar trends in our understanding of the gSAD through time, where Poisson log-normal gains importance and becomes the statistically best fit by the end of the time series. Illustrated here is the fit for Amphibia. The Chi-sqaure test was two-sided.

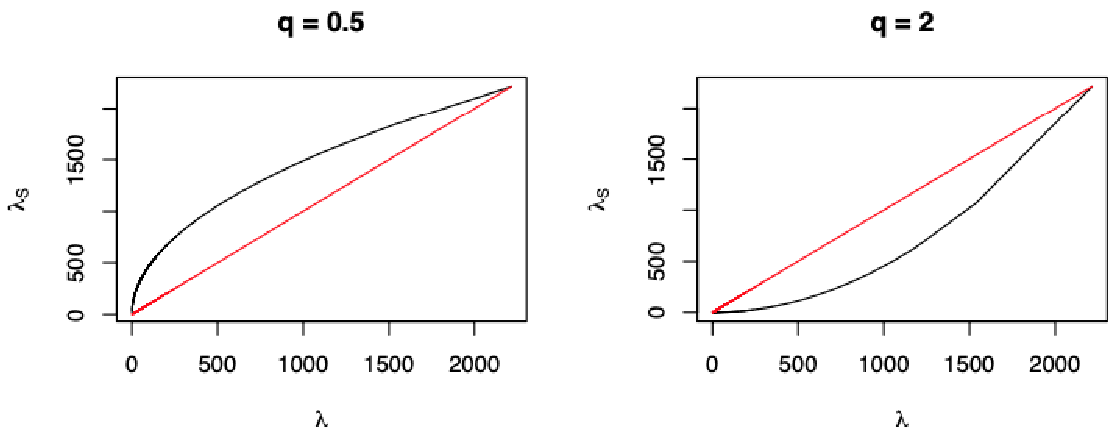

Fig. S25. Hypothetical relationship between observed abundances (as measured by number of occurrences in GBIF) and the real abundances of species. The observed abundances can be biased towards rare species (left) or biased towards common species (right). See methods for more details.

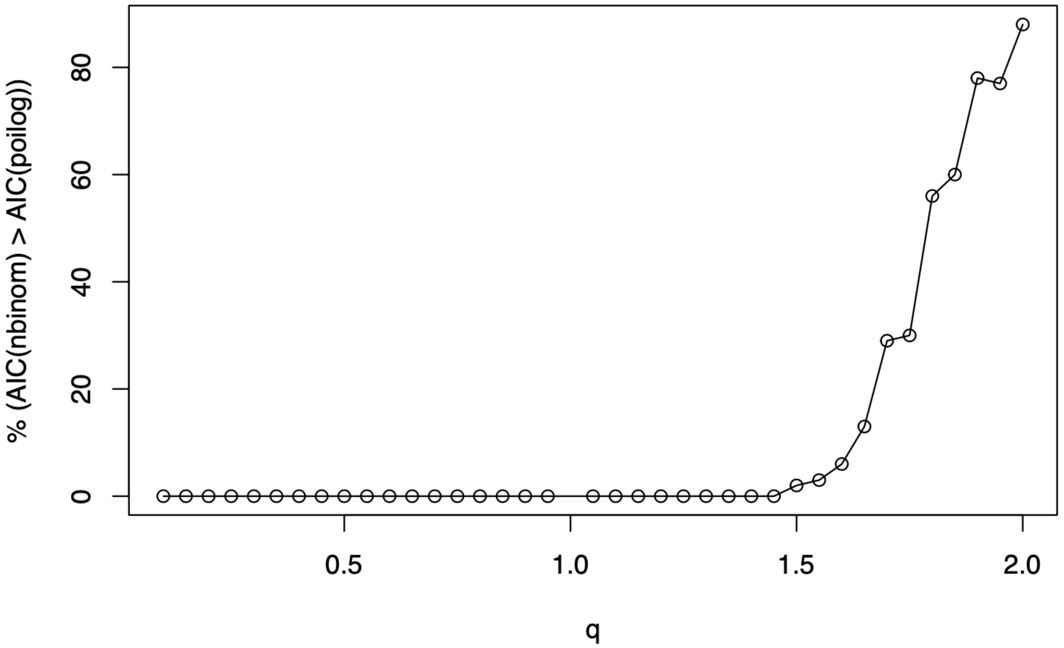

Fig. S26. Proportion of simulated communities for which the Poisson log-normal is a better fit than the negative binomial at different values of *q*, from extremely biased occurrences towards rare species (q=0.1) to extremely biased occurrences towards common species (q=2), for communities sampled from a gamma distribution with the best GBIF fit parameters for birds. Similar results occur when communities are sampled from a gamma distribution with the best fit parameters for mammals.

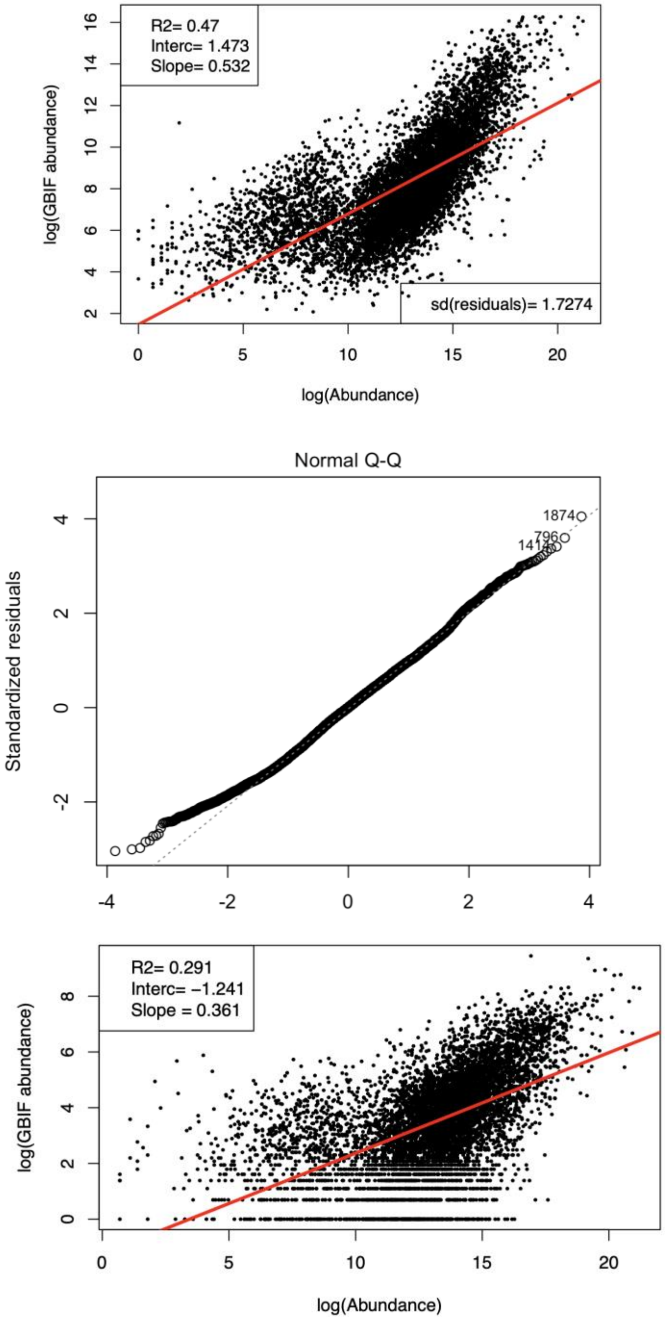

Fig. S27. Relationship between log number of GBIF occurrences and log estimated global abundance for birds. Top: linear regression plot for GBIF occurrences 1999-2019. Middle: Q-Q plot of the residuals 1999-2019. Bottom: linear regression plot for GBIF occurrences 1900-1919.

Movie S1-S39. Animation of the evolution of the gSAD, using 20-year rolling windows, for each of the 39 taxonomic classes included in the analysis. The video files are available here.

Table S1. The delta AIC values for the classes, comparing fitted models using AIC, for the 20-year rolling window model for year 2000. Models were compared using the AICtab function. Note that Insecta and Magnoliopsida are not shown here as the negative binomial models for these groups did not converge. The two classes that did not have a best delta AIC for Poisson log-normal are bolded.

Class	ΔAIC		
	Poisson log-normal	log-series	negative binomial
Actinopterygii	0.00	6232.85	6328.49
Amphibia	0.00	1204.72	1229.46
Anthocerotopsida	0.00	9.72	12.12
Anthozoa	0.00	535.50	552.83
Arachnida	0.00	4982.01	5062.16
Asteroidea	0.00	243.41	250.38
Aves	0.00	4612.94	4061.56
Bivalvia	0.00	1296.69	1314.52
Bryopsida	0.00	1587.99	1629.51
Cephalopoda	0.00	191.84	197.65
**Charophyceae**	**6.77**	**0.00**	**16.85**
Chilopoda	0.00	76.44	80.09
Chlorophyceae	0.00	66.95	70.53
Clitellata	0.00	412.87	422.84
Cycadopsida	0.00	92.74	79.35
Demospongiae	0.00	669.60	682.74
Echinoidea	0.00	106.65	111.03
Elasmobranchii	0.00	261.48	455.51
Entognatha	0.00	209.37	215.56
Florideophyceae	0.00	296.47	303.31
Gastropoda	0.00	10016.58	10163.06
Gymnolaemata	0.00	707.15	720.02
Hexanauplia	0.00	773.77	1306.95
Hydrozoa	0.00	321.36	330.08
Jungermanniopsida	0.00	895.91	951.78
Liliopsida	0.00	22913.68	26834.27
Lycopodiopsida	0.00	317.39	324.93
Malacostraca	0.00	5014.07	5081.55
Mammalia	0.00	1293.69	2152.80
Marchantiopsida	0.00	49.92	80.04
Maxillopoda	0.00	104.95	113.02
Ophiuroidea	0.00	276.64	283.31
Pinopsida	0.00	133.89	227.09
Polychaeta	0.00	1197.70	1226.94
Polyplacophora	0.00	77.42	81.05
Polypodiopsida	0.00	3028.83	3045.23
Reptilia	0.00	1522.17	1559.95
**Sphagnopsida**	**3.38**	**0.00**	**34.20**
Ulvophyceae	0.00	29.56	34.71
