# Supplementary figures and images for: Unveiling global species abundance distributions
Source: Nat Ecol Evol. 2023 Sep 4;7(10):1600–9. doi: 10.1038/s41559-023-02173-y (PMC10555817; doi:10.1038/s41559-023-02173-y)

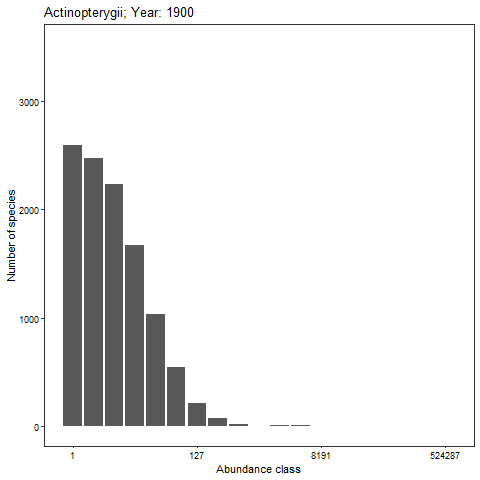

Supplement: Supplementary file 3 — Supplementary Videos 1–39. Animation of the evolution of the gSAD, using 20-year rolling windows, for each of the 39 taxonomic classes included in the analysis. [file 41559_2023_2173_MOESM3_ESM.zip › Supplementary_movies/Actinopterygii.gif]

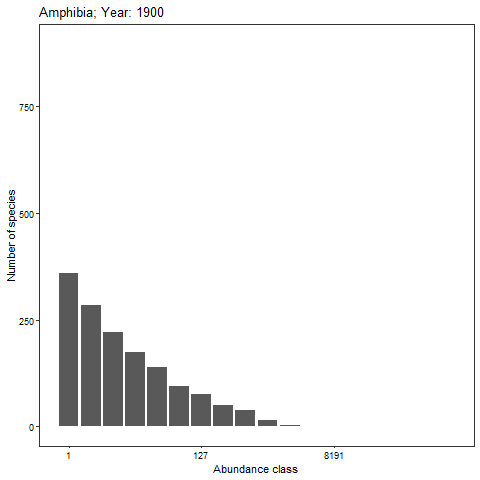

Supplement: Supplementary file 3 — Supplementary Videos 1–39. Animation of the evolution of the gSAD, using 20-year rolling windows, for each of the 39 taxonomic classes included in the analysis. [file 41559_2023_2173_MOESM3_ESM.zip › Supplementary_movies/Amphibia.gif]

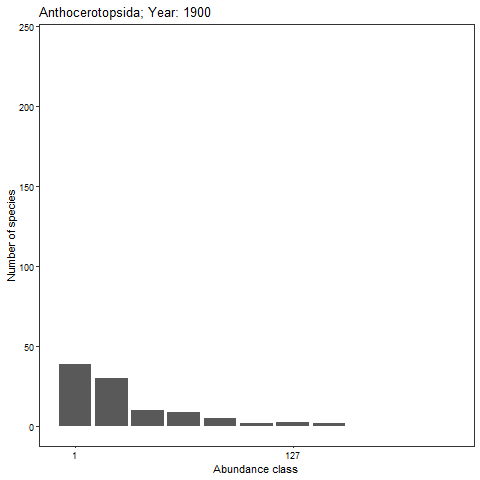

Supplement: Supplementary file 3 — Supplementary Videos 1–39. Animation of the evolution of the gSAD, using 20-year rolling windows, for each of the 39 taxonomic classes included in the analysis. [file 41559_2023_2173_MOESM3_ESM.zip › Supplementary_movies/Anthocerotopsida.gif]

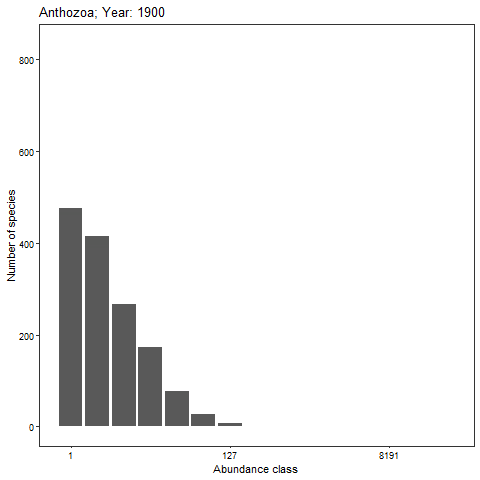

Supplement: Supplementary file 3 — Supplementary Videos 1–39. Animation of the evolution of the gSAD, using 20-year rolling windows, for each of the 39 taxonomic classes included in the analysis. [file 41559_2023_2173_MOESM3_ESM.zip › Supplementary_movies/Anthozoa.gif]

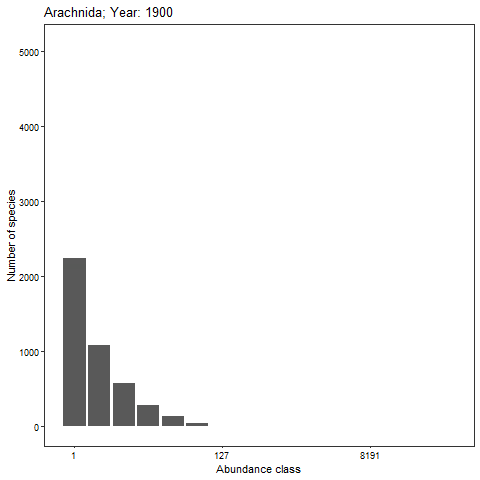

Supplement: Supplementary file 3 — Supplementary Videos 1–39. Animation of the evolution of the gSAD, using 20-year rolling windows, for each of the 39 taxonomic classes included in the analysis. [file 41559_2023_2173_MOESM3_ESM.zip › Supplementary_movies/Arachnida.gif]

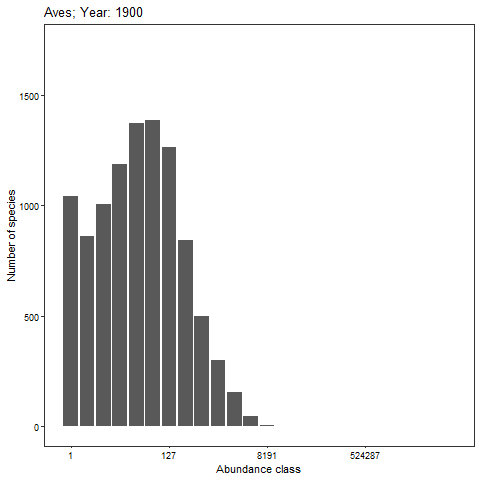

Supplement: Supplementary file 3 — Supplementary Videos 1–39. Animation of the evolution of the gSAD, using 20-year rolling windows, for each of the 39 taxonomic classes included in the analysis. [file 41559_2023_2173_MOESM3_ESM.zip › Supplementary_movies/Aves.gif]

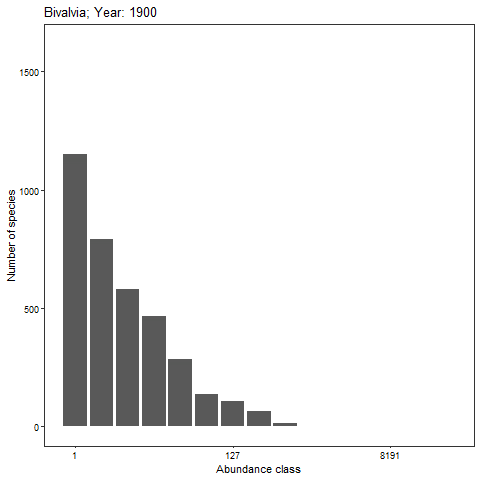

Supplement: Supplementary file 3 — Supplementary Videos 1–39. Animation of the evolution of the gSAD, using 20-year rolling windows, for each of the 39 taxonomic classes included in the analysis. [file 41559_2023_2173_MOESM3_ESM.zip › Supplementary_movies/Bivalvia.gif]

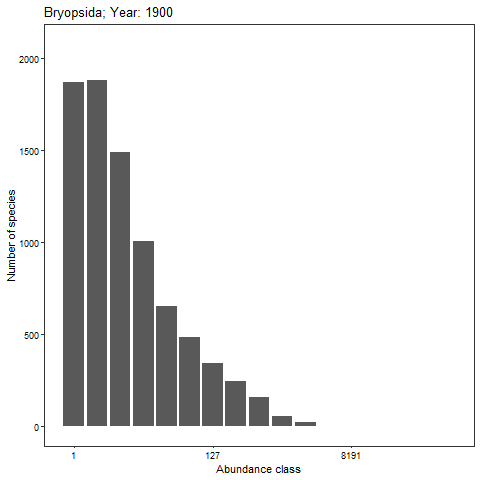

Supplement: Supplementary file 3 — Supplementary Videos 1–39. Animation of the evolution of the gSAD, using 20-year rolling windows, for each of the 39 taxonomic classes included in the analysis. [file 41559_2023_2173_MOESM3_ESM.zip › Supplementary_movies/Bryopsida.gif]

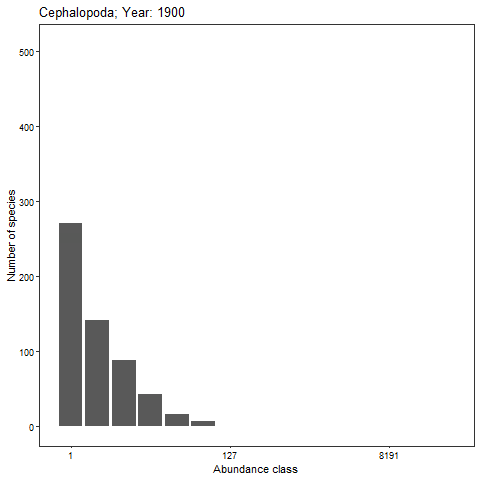

Supplement: Supplementary file 3 — Supplementary Videos 1–39. Animation of the evolution of the gSAD, using 20-year rolling windows, for each of the 39 taxonomic classes included in the analysis. [file 41559_2023_2173_MOESM3_ESM.zip › Supplementary_movies/Cephalopoda.gif]

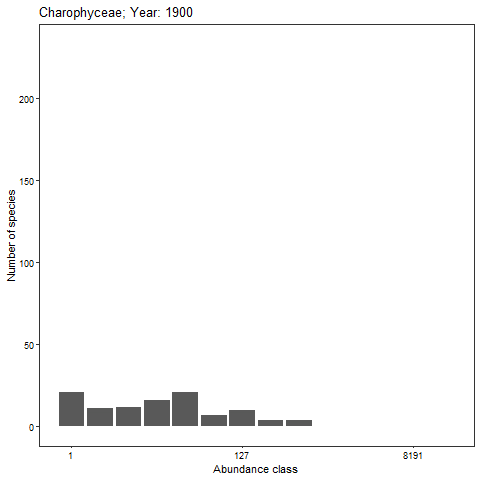

Supplement: Supplementary file 3 — Supplementary Videos 1–39. Animation of the evolution of the gSAD, using 20-year rolling windows, for each of the 39 taxonomic classes included in the analysis. [file 41559_2023_2173_MOESM3_ESM.zip › Supplementary_movies/Charophyceae.gif]

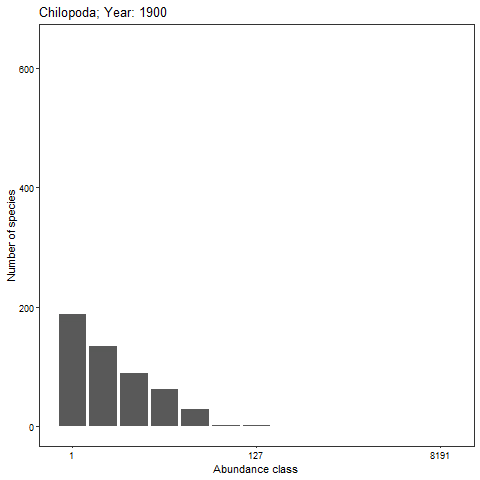

Supplement: Supplementary file 3 — Supplementary Videos 1–39. Animation of the evolution of the gSAD, using 20-year rolling windows, for each of the 39 taxonomic classes included in the analysis. [file 41559_2023_2173_MOESM3_ESM.zip › Supplementary_movies/Chilopoda.gif]

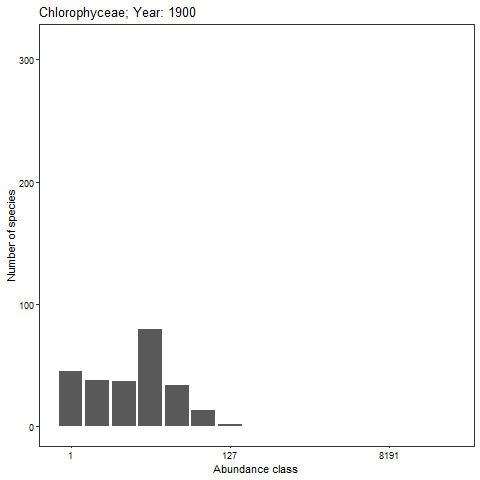

Supplement: Supplementary file 3 — Supplementary Videos 1–39. Animation of the evolution of the gSAD, using 20-year rolling windows, for each of the 39 taxonomic classes included in the analysis. [file 41559_2023_2173_MOESM3_ESM.zip › Supplementary_movies/Chlorophyceae.gif]

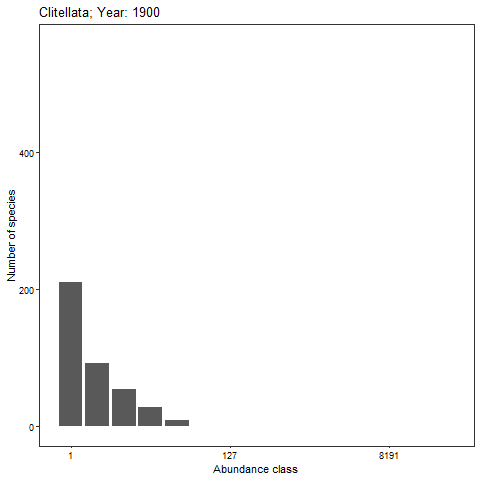

Supplement: Supplementary file 3 — Supplementary Videos 1–39. Animation of the evolution of the gSAD, using 20-year rolling windows, for each of the 39 taxonomic classes included in the analysis. [file 41559_2023_2173_MOESM3_ESM.zip › Supplementary_movies/Clitellata.gif]

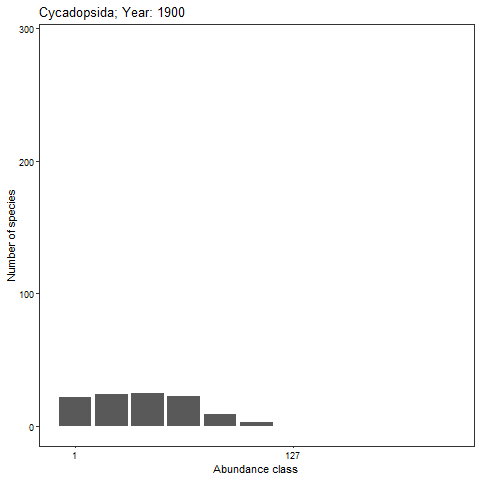

Supplement: Supplementary file 3 — Supplementary Videos 1–39. Animation of the evolution of the gSAD, using 20-year rolling windows, for each of the 39 taxonomic classes included in the analysis. [file 41559_2023_2173_MOESM3_ESM.zip › Supplementary_movies/Cycadopsida.gif]

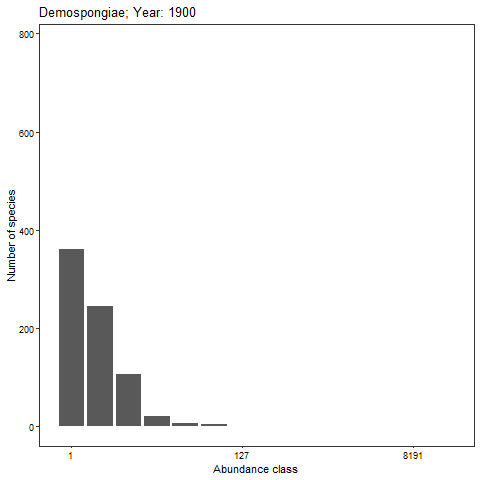

Supplement: Supplementary file 3 — Supplementary Videos 1–39. Animation of the evolution of the gSAD, using 20-year rolling windows, for each of the 39 taxonomic classes included in the analysis. [file 41559_2023_2173_MOESM3_ESM.zip › Supplementary_movies/Demospongiae.gif]

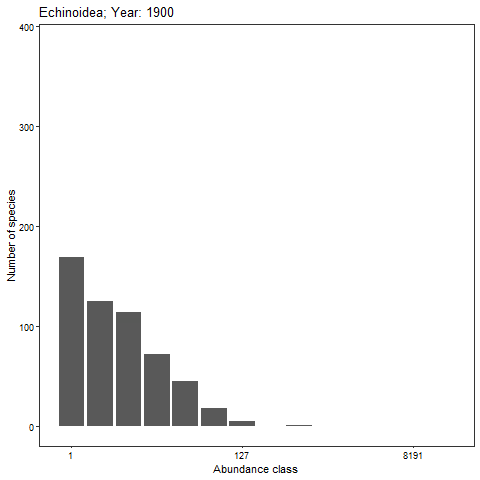

Supplement: Supplementary file 3 — Supplementary Videos 1–39. Animation of the evolution of the gSAD, using 20-year rolling windows, for each of the 39 taxonomic classes included in the analysis. [file 41559_2023_2173_MOESM3_ESM.zip › Supplementary_movies/Echinoidea.gif]

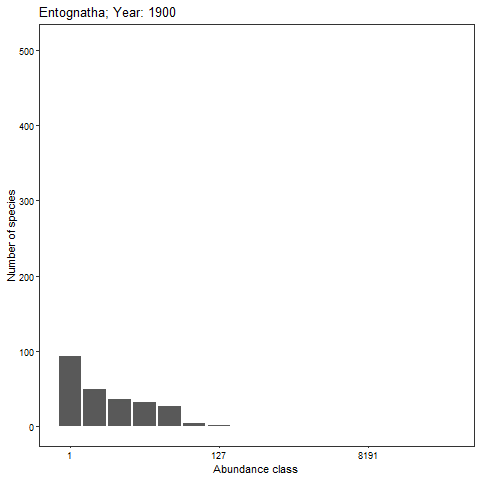

Supplement: Supplementary file 3 — Supplementary Videos 1–39. Animation of the evolution of the gSAD, using 20-year rolling windows, for each of the 39 taxonomic classes included in the analysis. [file 41559_2023_2173_MOESM3_ESM.zip › Supplementary_movies/Entognatha.gif]

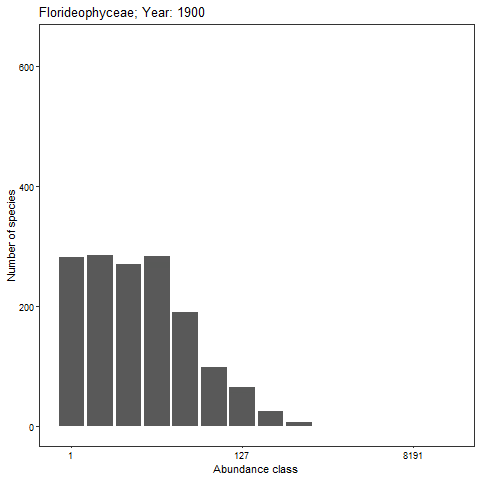

Supplement: Supplementary file 3 — Supplementary Videos 1–39. Animation of the evolution of the gSAD, using 20-year rolling windows, for each of the 39 taxonomic classes included in the analysis. [file 41559_2023_2173_MOESM3_ESM.zip › Supplementary_movies/Florideophyceae.gif]

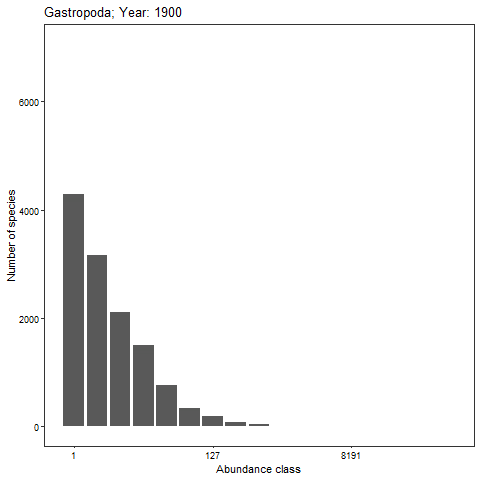

Supplement: Supplementary file 3 — Supplementary Videos 1–39. Animation of the evolution of the gSAD, using 20-year rolling windows, for each of the 39 taxonomic classes included in the analysis. [file 41559_2023_2173_MOESM3_ESM.zip › Supplementary_movies/Gastropoda.gif]

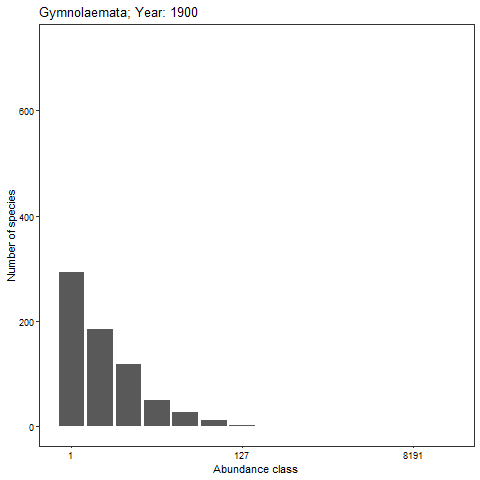

Supplement: Supplementary file 3 — Supplementary Videos 1–39. Animation of the evolution of the gSAD, using 20-year rolling windows, for each of the 39 taxonomic classes included in the analysis. [file 41559_2023_2173_MOESM3_ESM.zip › Supplementary_movies/Gymnolaemata.gif]

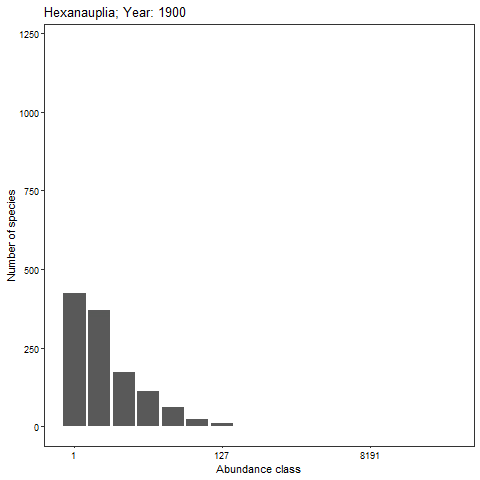

Supplement: Supplementary file 3 — Supplementary Videos 1–39. Animation of the evolution of the gSAD, using 20-year rolling windows, for each of the 39 taxonomic classes included in the analysis. [file 41559_2023_2173_MOESM3_ESM.zip › Supplementary_movies/Hexanauplia.gif]

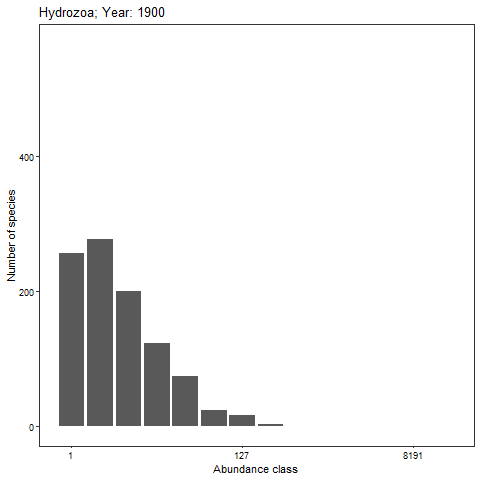

Supplement: Supplementary file 3 — Supplementary Videos 1–39. Animation of the evolution of the gSAD, using 20-year rolling windows, for each of the 39 taxonomic classes included in the analysis. [file 41559_2023_2173_MOESM3_ESM.zip › Supplementary_movies/Hydrozoa.gif]

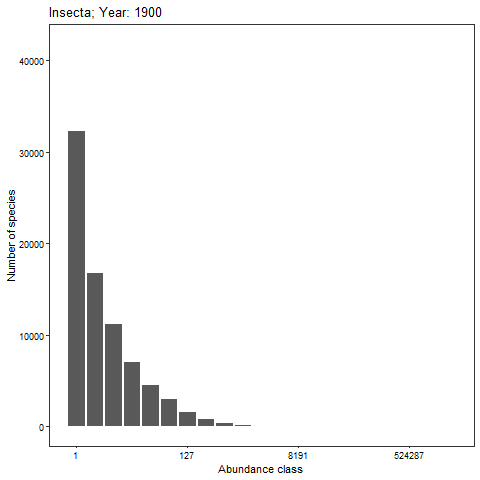

Supplement: Supplementary file 3 — Supplementary Videos 1–39. Animation of the evolution of the gSAD, using 20-year rolling windows, for each of the 39 taxonomic classes included in the analysis. [file 41559_2023_2173_MOESM3_ESM.zip › Supplementary_movies/Insecta.gif]

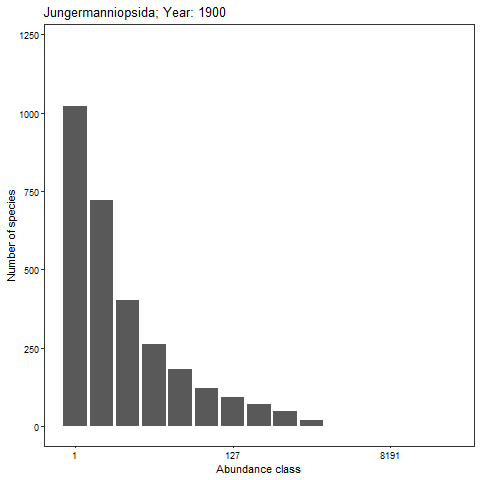

Supplement: Supplementary file 3 — Supplementary Videos 1–39. Animation of the evolution of the gSAD, using 20-year rolling windows, for each of the 39 taxonomic classes included in the analysis. [file 41559_2023_2173_MOESM3_ESM.zip › Supplementary_movies/Jungermanniopsida.gif]

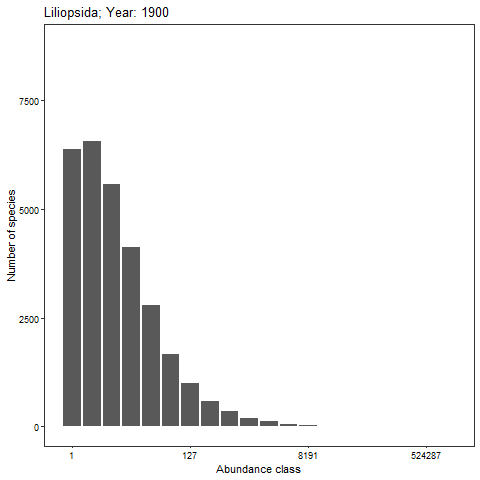

Supplement: Supplementary file 3 — Supplementary Videos 1–39. Animation of the evolution of the gSAD, using 20-year rolling windows, for each of the 39 taxonomic classes included in the analysis. [file 41559_2023_2173_MOESM3_ESM.zip › Supplementary_movies/Liliopsida.gif]

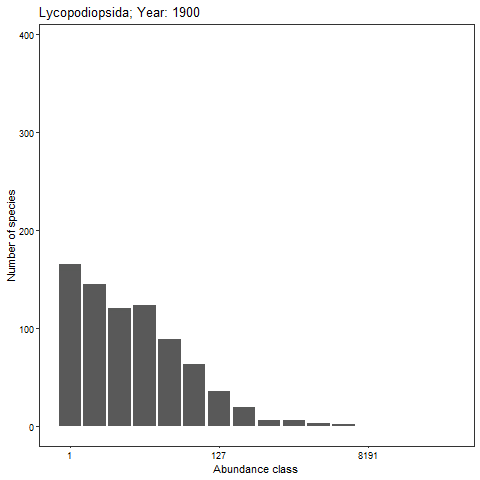

Supplement: Supplementary file 3 — Supplementary Videos 1–39. Animation of the evolution of the gSAD, using 20-year rolling windows, for each of the 39 taxonomic classes included in the analysis. [file 41559_2023_2173_MOESM3_ESM.zip › Supplementary_movies/Lycopodiopsida.gif]

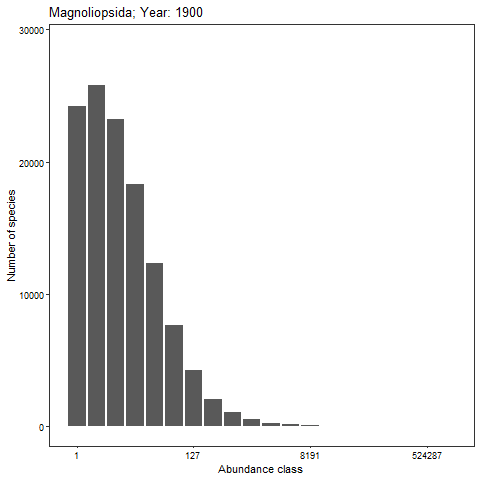

Supplement: Supplementary file 3 — Supplementary Videos 1–39. Animation of the evolution of the gSAD, using 20-year rolling windows, for each of the 39 taxonomic classes included in the analysis. [file 41559_2023_2173_MOESM3_ESM.zip › Supplementary_movies/Magnoliopsida.gif]

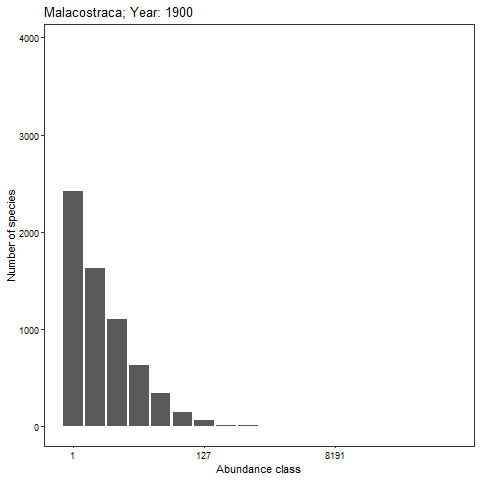

Supplement: Supplementary file 3 — Supplementary Videos 1–39. Animation of the evolution of the gSAD, using 20-year rolling windows, for each of the 39 taxonomic classes included in the analysis. [file 41559_2023_2173_MOESM3_ESM.zip › Supplementary_movies/Malacostraca.gif]

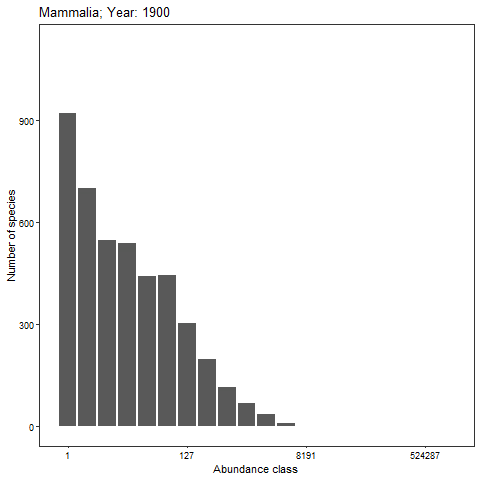

Supplement: Supplementary file 3 — Supplementary Videos 1–39. Animation of the evolution of the gSAD, using 20-year rolling windows, for each of the 39 taxonomic classes included in the analysis. [file 41559_2023_2173_MOESM3_ESM.zip › Supplementary_movies/Mammalia.gif]

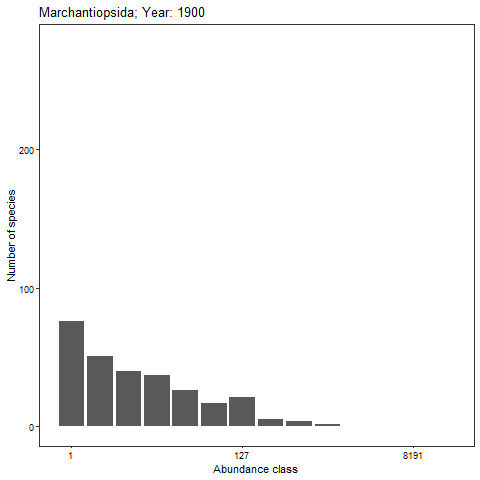

Supplement: Supplementary file 3 — Supplementary Videos 1–39. Animation of the evolution of the gSAD, using 20-year rolling windows, for each of the 39 taxonomic classes included in the analysis. [file 41559_2023_2173_MOESM3_ESM.zip › Supplementary_movies/Marchantiopsida.gif]

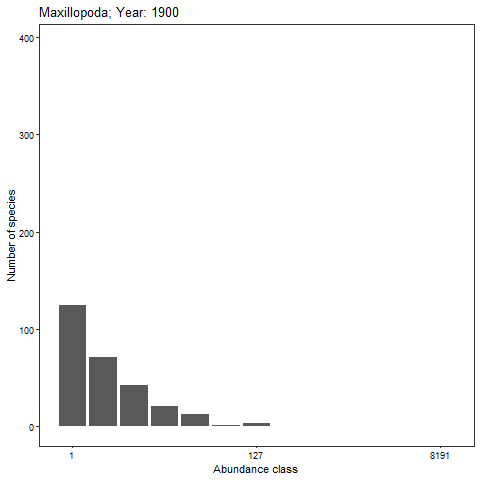

Supplement: Supplementary file 3 — Supplementary Videos 1–39. Animation of the evolution of the gSAD, using 20-year rolling windows, for each of the 39 taxonomic classes included in the analysis. [file 41559_2023_2173_MOESM3_ESM.zip › Supplementary_movies/Maxillopoda.gif]

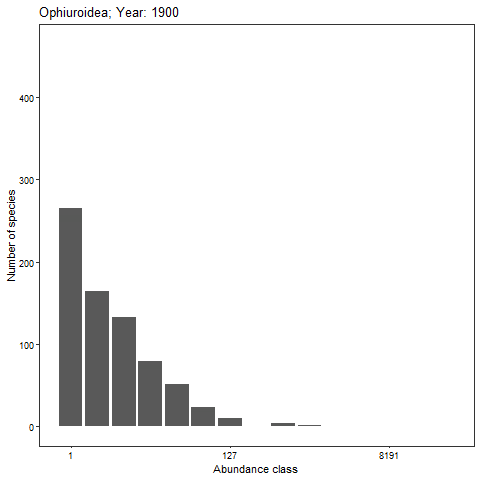

Supplement: Supplementary file 3 — Supplementary Videos 1–39. Animation of the evolution of the gSAD, using 20-year rolling windows, for each of the 39 taxonomic classes included in the analysis. [file 41559_2023_2173_MOESM3_ESM.zip › Supplementary_movies/Ophiuroidea.gif]

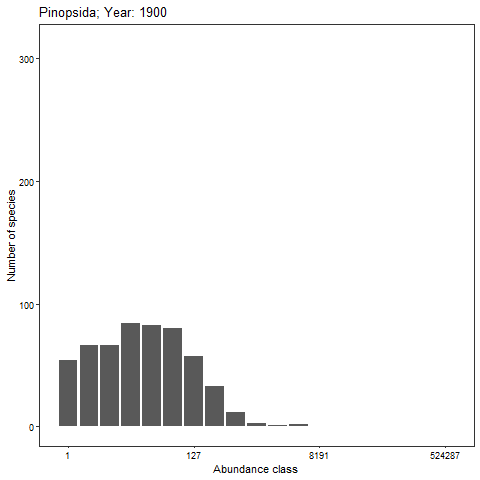

Supplement: Supplementary file 3 — Supplementary Videos 1–39. Animation of the evolution of the gSAD, using 20-year rolling windows, for each of the 39 taxonomic classes included in the analysis. [file 41559_2023_2173_MOESM3_ESM.zip › Supplementary_movies/Pinopsida.gif]

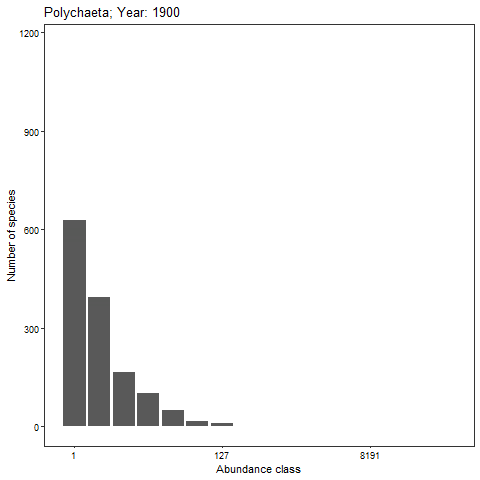

Supplement: Supplementary file 3 — Supplementary Videos 1–39. Animation of the evolution of the gSAD, using 20-year rolling windows, for each of the 39 taxonomic classes included in the analysis. [file 41559_2023_2173_MOESM3_ESM.zip › Supplementary_movies/Polychaeta.gif]

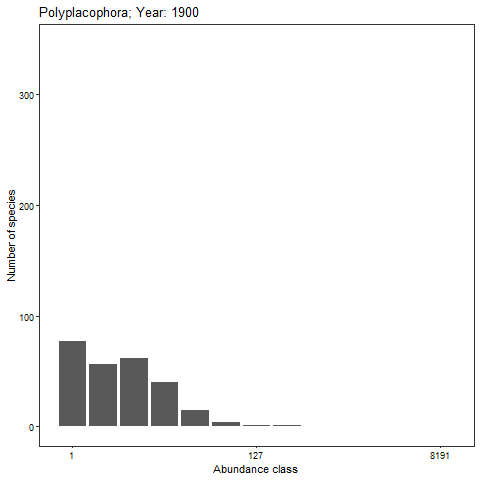

Supplement: Supplementary file 3 — Supplementary Videos 1–39. Animation of the evolution of the gSAD, using 20-year rolling windows, for each of the 39 taxonomic classes included in the analysis. [file 41559_2023_2173_MOESM3_ESM.zip › Supplementary_movies/Polyplacophora.gif]

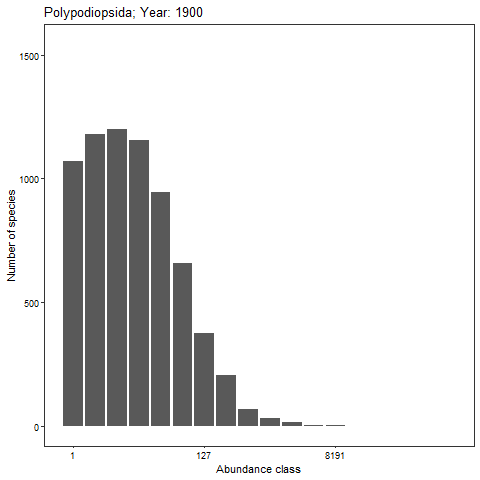

Supplement: Supplementary file 3 — Supplementary Videos 1–39. Animation of the evolution of the gSAD, using 20-year rolling windows, for each of the 39 taxonomic classes included in the analysis. [file 41559_2023_2173_MOESM3_ESM.zip › Supplementary_movies/Polypodiopsida.gif]

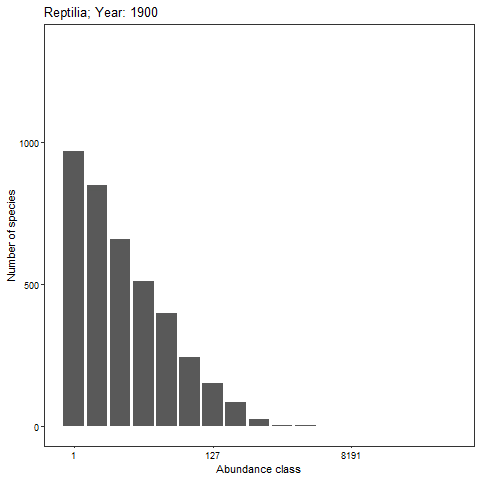

Supplement: Supplementary file 3 — Supplementary Videos 1–39. Animation of the evolution of the gSAD, using 20-year rolling windows, for each of the 39 taxonomic classes included in the analysis. [file 41559_2023_2173_MOESM3_ESM.zip › Supplementary_movies/Reptilia.gif]

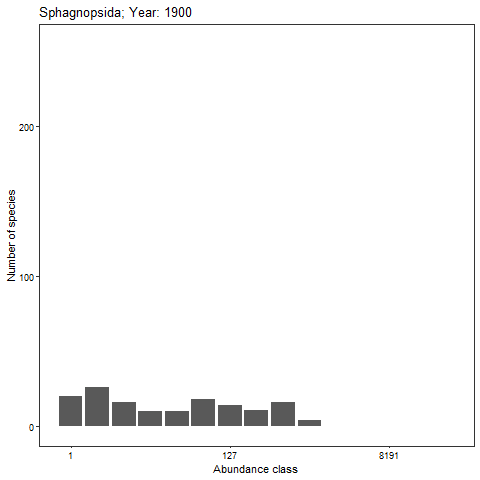

Supplement: Supplementary file 3 — Supplementary Videos 1–39. Animation of the evolution of the gSAD, using 20-year rolling windows, for each of the 39 taxonomic classes included in the analysis. [file 41559_2023_2173_MOESM3_ESM.zip › Supplementary_movies/Sphagnopsida.gif]

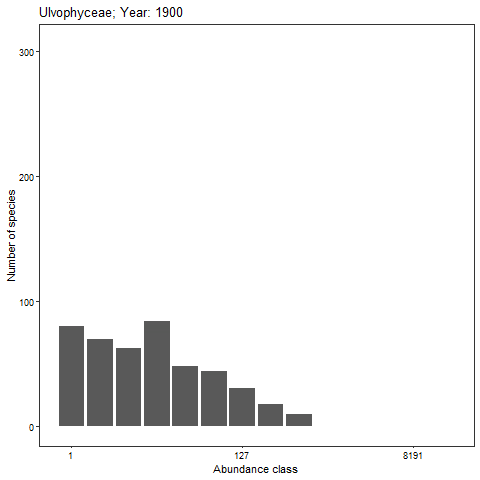

Supplement: Supplementary file 3 — Supplementary Videos 1–39. Animation of the evolution of the gSAD, using 20-year rolling windows, for each of the 39 taxonomic classes included in the analysis. [file 41559_2023_2173_MOESM3_ESM.zip › Supplementary_movies/Ulvophyceae.gif]
